# Leveraging machine learning for taxonomic classification of emerging astroviruses

**DOI:** 10.3389/fmolb.2023.1305506

**Published:** 2024-01-11

**Authors:** Fatemeh Alipour, Connor Holmes, Yang Young Lu, Kathleen A. Hill, Lila Kari

**Affiliations:** ^1^ School of Computer Science, University of Waterloo, Waterloo, ON, Canada; ^2^ Department of Biology, University of Western Ontario, London, ON, Canada

**Keywords:** machine learning, viral classification and clustering, family *Astroviridae*, *Avastrovirus*, *Mamastrovirus*, alignment-free classification, genomic signature, k-mer frequency

## Abstract

Astroviruses are a family of genetically diverse viruses associated with disease in humans and birds with significant health effects and economic burdens. Astrovirus taxonomic classification includes two genera, *Avastrovirus* and *Mamastrovirus.* However, with next-generation sequencing, broader interspecies transmission has been observed necessitating a reexamination of the current host-based taxonomic classification approach. In this study, a novel taxonomic classification method is presented for emergent and as yet unclassified astroviruses, based on whole genome sequence *k*-mer composition in addition to host information. An optional component responsible for identifying recombinant sequences was added to the method’s pipeline, to counteract the impact of genetic recombination on viral classification. The proposed three-pronged classification method consists of a supervised machine learning method, an unsupervised machine learning method, and the consideration of host species. Using this three-pronged approach, we propose genus labels for 191 as yet unclassified astrovirus genomes. Genus labels are also suggested for an additional eight as yet unclassified astrovirus genomes for which incompatibility was observed with the host species, suggesting cross-species infection. Lastly, our machine learning-based approach augmented by a principal component analysis (PCA) analysis provides evidence supporting the hypothesis of the existence of human astrovirus (*HAstV*) subgenus of the genus *Mamastrovirus*, and a goose astrovirus (*GoAstV*) subgenus of the genus *Avastrovirus*. Overall, this multipronged machine learning approach provides a fast, reliable, and scalable prediction method of taxonomic labels, able to keep pace with emerging viruses and the exponential increase in the output of modern genome sequencing technologies.

## 1 Introduction

Astroviruses are a genetically diverse virus family notably responsible for the second most common cause of nosocomial diarrhea following rotaviruses (Meyer et al., 2015), as well as substantial economic losses in the poultry industry (Karlsson et al., 2015; Li et al., 2023). Also, astrovirus infection has been associated with encephalitis and meningitis in immunocompromised patients (Vu et al., 2017) and astrovirus infection has been shown to be present in the brains of some mammals (Chae et al., 2023). According to several studies (Resque et al., 2007; Meyer et al., 2015; Keita et al., 2023), the prevalence of astroviruses among human populations ranges from 2% to 9% at any given time. In developing countries, this percentage can be significantly higher, affecting up to 30% of the population (De Benedictis et al., 2011). Infants between 3 and 8 months of age (Herrmann et al., 1991; Shastri et al., 1998; Dennehy et al., 2001; Jeong et al., 2012; Chhabra et al., 2013), and the elderly (Babkin et al., 2012) along with immunocompromised patients are primarily infected (Grohmann et al., 1993; Palombo and Bishop, 1996; Liste et al., 2000). Outbreaks have been reported for immunocompetent adults (Midthun et al., 1993; Oishi et al., 1994; Jarchow-Macdonald et al., 2015). Astrovirus transmission occurs exclusively through the fecal-oral route (Lefkowitz et al., 2017), with notable interspecies transmission (De Benedictis et al., 2011). Astrovirus genetic diversity is linked to the proposed replication via the class III PI3K pathway during autophagy (Bub et al., 2023) and genetic recombination associated with cross-species transmission through abiotic vectors such as drinking water, sewage, and other contaminated systems (Abad et al., 1997; Le Cann et al., 2004). The increasing interspecies transmission enhances the risk of extraintestinal infections in humans as reported in animal populations (Qureshi et al., 2023). Genetic recombination elevates genetic diversity in the context of a concurrent multiplicity of infections for viruses of different genera given cross-species transmissions.

The International Committee on the Taxonomy of Viruses (ICTV) structures the family *Astroviridae* into two genera, *Avastrovirus* and *Mamastrovirus* (Lefkowitz et al., 2017), and determines taxonomic classification by defining species intragroups as strains with a minimum amino acid identity of 75% in the open reading frame 2 (ORF2) region. The two genera include many host-associated astroviruses and the number of known animal hosts has reached over 160, spanning 13 classes of organisms. Next-generation sequencing continues to achieve rapid detection of new astroviruses and the identification of new host species (Vu et al., 2017; Zheng et al., 2017), with a steadily increasing number of astrovirus genomes on the National Center for Biotechnology Information (NCBI) awaiting classification at the genus level.

Urgency and need to refine the taxonomy of family *Astroviridae* is accelerated by reports of astrovirus recombination during concomitant infections (Pantin-Jackwood et al., 2012; Lefkowitz et al., 2017), including some involving *HAstV*s (Vu et al., 2017). Also, avian and mammalian astrovirus species have been found in nonhuman primates (Cortez et al., 2017), and inter-species crossover between humans and various animals, such as felines, cats, pigs, California sea lions, dogs, sheep, and turkeys have been identified (Jiang et al., 1993; Meliopoulos et al., 2014; Karlsson et al., 2015; Cortez et al., 2017). These observations complicate a taxonomic classification based solely on host species and nucleotide sequence identity. Moreover, with the emergence of interspecies transmission, confusion has arisen in classifying astroviruses based on their origins. Due to classification at the species level based on sequence identity, some inter-cluster species of different genera, namely, *HAstV*s, show more relatedness than those of the same intra-cluster genus (Jiang et al., 1993). This has led to a call for standardized methods of classification for family *Astroviridae* (Cortez et al., 2017).

Recent years have seen a rapid growth in the volume of accessible genomic data, due to notable advancements in next-generation sequencing (NGS) technologies and a reduction in sequencing costs (Schwende and Pham, 2014). Consequently, there is an increasing demand for computationally efficient and scalable methods to handle large genomic datasets (Shendure et al., 2004; Katz et al., 2022). Earlier attempts to tackle genomic classification/clustering problems can be categorized into two approaches: “alignment-based” and “alignment-free” methods. The high computational cost and the reliance on sequence homology of alignment-based techniques make alignment-free methods a more suitable choice for addressing the virus classification problem. Consequently, a multitude of alignment-free classification (Solis-Reyes et al., 2018; Fabijańska and Grabowski, 2019; Randhawa et al., 2019; Jiang et al., 2023) and clustering methods (Girgis, 2022; Millán Arias et al., 2022; Millan Arias et al., 2023) suitable for viral genomic sequence datasets have emerged, and initial studies demonstrated their effectiveness and scalability compared to traditional alignment-based methods (Thompson et al., 1994; Edgar, 2004).

This paper presents a novel machine-learning classification method hereafter called the Three-Pronged Classification Method (3PCM) to classify astrovirus sequences that are as yet unclassified. The method utilizes the primary sequence composition of the entire genome in the form of *k*-mer frequency vectors, where *k* is set to 6. In this paper, *k* = 6 was empirically found to achieve the best balance between accuracy and computational complexity. An initial optional component was incorporated into 3PCM’s pipeline to detect potential recombinant sequences and exclude them from the analysis. This step aims to prevent any noise caused by inter-species crossover, which could otherwise confound machine-learning models. 3PCM consists of three main components: Prong 1 (a supervised classification method utilizing Quadratic SVM), Prong 2 (an unsupervised clustering technique based on K-means++); and Prong 3 (the identification of host labels at the class level from relevant literature for the as yet unclassified viral sequences). In this paper, taxonomic classification was suggested when all three prongs of 3PCM agreed on a taxonomic label. When Prong 1 and Prong 2 concurred on a classification that differed from Prong 3, a taxonomic classification was suggested, subject to independent confirmation.

The design of 3PCM utilizes genome composition information from astrovirus sequences with known taxonomic labels to classify/cluster astrovirus sequences with mammalian or avian hosts that are as yet unclassified. Although the default output of 3PCM is based on the consensus prediction of the three prongs, the individual prongs can be used independently in cases where one or two prongs are not applicable or do not agree. For instance, Prong 1 is not suitable for classifying as yet unclassified astrovirus sequences with non-mammalian non-avian hosts, due to the absence of ground truth labels which are necessary for training a supervised model. In such a situation, Prong 2 can be used in conjunction with Prong 3 to investigate the classification of the sequences. In this and other scenarios, other analyses such as genome composition analysis can be employed to validate the results.

The main contributions of this paper are:

• Proposing genus labels (*Mamastrovirus* or *Avastrovirus*) for 191 as yet unclassified astrovirus genome sequences for which the results of Prongs 1, 2, and 3 all agree.
• Suggesting genus labels (*Mamastrovirus* or *Avastrovirus*) for 8 additional as yet unclassified astrovirus genome sequences, for which incompatibility was observed between the taxonomic label proposed by Prong 1 and Prong 2, and the host label provided by Prong 3. This may be due to cross-species transmission, and further investigation is needed to resolve the contrasting labels associated with these sequences.
• Providing evidence supporting the hypothesis of the existence of a human astrovirus subgenus of the genus *Mamastrovirus* and a goose astrovirus subgenus of the genus *Avastrovirus*, through the application of the proposed machine learning-based approach, enhanced by a principal component analysis (PCA) of the sequence composition.

Overall, this multipronged machine learning approach provides a fast, reliable, and scalable prediction method of taxonomic labels, able to keep pace with emerging viruses and the exponential increase in the output of modern genome sequencing technologies.

## 2 Materials and methods

The first part of this section, Materials, provides an overview of the dataset used in this study. The second subsection, Methods, describes the technical and implementation details of three prongs of the proposed classification method. Moreover, the evaluation metrics used to evaluate the proposed methodology will be discussed throughout the Methods section.

### 2.1 Materials: datasets

The dataset used in this study consists of RNA sequences from the viral family, *Astroviridae* downloaded from the NCBI database. In the RNA sequence, Ns replaced all sequence characters other than adenine (A), cytosine (C), guanine (G), and uracil (U). The N in an RNA sequence means that any of the four bases could occupy the position in question. All sequences were uploaded to a folder in Genbank. These were then exported as a single multifasta file for further testing.

A total of 1,039 sequences from the family *Astroviridae* were downloaded from the NCBI database on 27th July 2022. The sequences included in this study were between 5 and 10 kbps in length. The host for each virus sequence was identified from the literature where a publication was available. In the absence of published records, the organism listed in the NCBI database submission was considered the host. We excluded 47 out of 1,039 sequences from our analysis due to the lack of information regarding the host of the virus given collection from sewage, rivers, and streams. Patent sequences were also excluded. Following the removal of these sequences, 992 sequences were used in this study as the primary dataset (Dataset 1, described in Table 1). Among the 992 sequences in this dataset, 308 are as yet unclassified at the genus level. The individual host species were ascribed to their respective class and genus. The final dataset contains Astrovirus genomes found in 13 unique host classes and 96 unique host genera.

**TABLE 1:** Description of Dataset 1 containing 992 viral genomes belonging to the family *Astroviridae*.

Genus	No. of sequences	Min. sequence length (bp)	Avg. sequence length (bp)	Max. sequence length (bp)
*Avastrovirus*	213	5,130	7,146	7,799
*Mamastrovirus*	471	5,003	6,395	7,353
Unknown	308	5,030	6,536	8,840
All/Average	992	5,003	6,600	8,840

*Avastrovirus* and *Mamastrovirus* are two genera currently designated within family *Astroviridae*. Of the 992 sequences, 308 lack a genus label as they have as yet not been classified at this taxonomic level.

In addition to Dataset 1, two other subsets of this dataset are used throughout the paper, as described below. Dataset 2 (described in Table 2) comprises the 684 genomes in Dataset 1 that belong to either *Avastrovirus* or *Mamastrovirus* genus. Dataset 2 was used both as the main training dataset and as the dataset employed for determining different parameters of the proposed three-pronged classification method.

**TABLE 2:** Description of Dataset 2, a subset of Dataset 1 consisting of sequences with available ground truth.

Genus	No. of sequences	Min. sequence length (bp)	Avg. sequence length (bp)	Max. sequence length (bp)
*Avastrovirus*	213	5,130	7,146	7,799
*Mamastrovirus*	471	5,003	6,395	7,353
All/Average	684	5,003	6,629	7,799

There are 684 sequences in Dataset 2 which belong to one of the two established genera of this viral family, *Avastrovirus* and *Mamastrovirus.*

With a goal to predict genus level labels for the 308 as yet unclassified sequences, we investigated the current information about these sequences, that is, their hosts. Please see Supplementary Material S1 (Analysis of Astroviruses of Unknown Genus Label), for the distribution of hosts for all the 308 Astrovirus genomes with unknown genus level labels. For purposes described in the Results section, Dataset 3 was created, consisting of 187 astrovirus genomes in Dataset 1 with unknown genus level labels and mammalian hosts, and 42 astrovirus genomes in Dataset 1 with unknown genus level labels and avian hosts (see Table 3).

**TABLE 3:** Description of Dataset 3, consisting of 187 astrovirus genomes in Dataset 1 that are as yet unclassified (unknown genus) and have a mammalian host, and 42 astrovirus genomes in Dataset 1 that are as yet unclassified (unknown genus) and have an avian host.

Genus	Host	No. of sequences	Min. sequence length (bp)	Avg. sequence length (bp)	Max. sequence length (bp)
Unknown	Mammalia	187	5,209	6,348	7,426
Unknown	Aves	42	5,084	6,806	8,417
All/Average	—	229	5,084	6,432	8,417

### 2.2 Methods

#### 2.2.1 Overview of the methodological pipeline

We herein propose a three-pronged classification method (3PCM) for the taxonomic classification of emergent but as yet unclassified astrovirus sequences.

**FIGURE 1:**
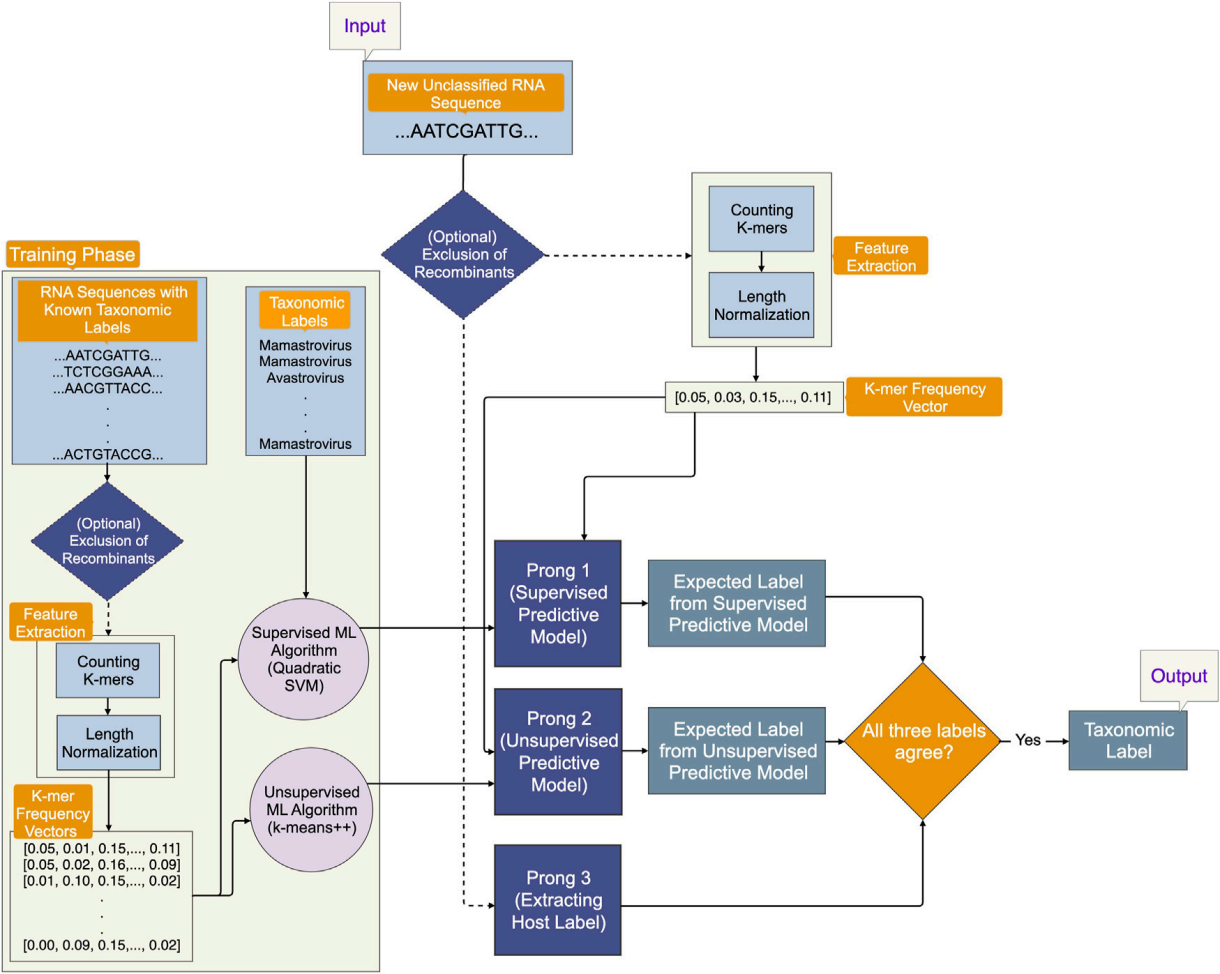
An overview of the proposed Three-Pronged Classification Method (3PCM). The input is an as yet unclassified genomic sequence. As an initial elective step, a component within the pipeline gives the option to eliminate recombinant sequences from the dataset. Prong 1 employs a supervised predictive model trained on genomic sequences with known taxonomic labels. Prong 2 uses an unsupervised predictive model trained on the same genomic sequences, but it does not use their taxonomic labels for training. Prong 3 uses the host label of the input genomic sequence. All three prongs of 3PCM must agree in their prediction, in order to produce a suggested taxonomic label.

An optional initial component of 3PCM aims to eliminate recombinant sequences from the training and testing datasets, for scenarios where their presence may confound the machine learning process. The main methodological pipeline consists of three prongs, as illustrated in Figure 1:

1. Prong 1 (supervised learning): training a classification model using the whole genome sequence for astroviruses with known taxonomic classification in the training phase and leveraging the trained predictive model to predict the labels of as yet unclassified astroviruses in the testing phase.
2. Prong 2 (unsupervised learning): training a clustering model using the whole genomes of astroviruses in the training phase and using the trained predictive model to predict taxonomic labels of as yet unclassified astroviruses in the testing phase. Taxonomic labels are not used in the training phase, therefore, this model is less vulnerable to inaccuracies of current taxonomic labels and classifications.
3. Prong 3 (identifying host label): identifying the class label (Mammalia, Aves, etc.) of the host from which the as yet unclassified viral sample was obtained.

In the event that the predictions of all three prongs agree, taxonomic labels are proposed for as yet unclassified sequences. If there is agreement only between Prong 1 and Prong 2, taxonomic labels are proposed with recommendations for further investigation.

#### 2.2.2 An optional initial component of eliminating recombinant sequences

3PCM can be used both with comprehensive datasets and with versions of those datasets where known or presumed recombinant sequences have been removed. This option was added because astroviruses exhibit genetic diversity in part through recombination, which is further complicated by concurrent infections with multiple astrovirus taxa and recombination with up to three genomes at once (Wei et al., 2023). The nature of this recombination significantly enhances the genetic diversity of astroviruses and hinders the reconstruction of astrovirus evolutionary history. To evaluate the impact of recombinant genomes on machine learning-based classification methods, the aligned sequences of this viral family were uploaded to the Recombination Detection Program *RDP*4 (Martin et al., 2015). *RDP*4 employed various tools such as *RDP*, *GENECONV*, *BOOTSCAN*, *MaxChi*, *SiScan*, *CHIMEARA*, and *TOPAL* to assess all possible sequence triplets and determine the recombinant sequence, major parent, and minor parent involved. In this study, the only recombination events that were considered were those where the parents were identified by two or more of the seven aforementioned tools, with a level of significance *p* < 0.05 (statistically significant). For further information please refer to Supplementary Material S2 (Identification and Analysis of Candidate Recombinant Astrovirus Genome Sequences).

#### 2.2.3 Prong 1: supervised machine learning

The first prong makes use of existing and established knowledge regarding astroviruses to train a supervised machine learning model based on the complete genomes of astroviruses with known taxonomic labels (Dataset 2) in the training phase. We then used the trained model to predict the labels of as yet unclassified astroviruses (Dataset 3) during the testing phase.

The features used in the supervised learning methods in this study were the *k*-mer frequency vector of each astrovirus genome. The *k*-mers containing an *N* (not specifically one of the four bases in RNA) were not included. The performance of Prong 1 for values of *k* in the range [1, 9] in terms of classification accuracy and running time can be found in Supplementary Material S3 (Performance Results of 3PCM Using Different Classification/Clustering Algorithms). The value *k* = 6 was empirically found to achieve the best balance between accuracy and computational complexity for the datasets and computational experiments in this paper. To avoid potential effects of sequence length variation (astrovirus genome lengths range between 5,003 and 8,840 bp), the feature vectors were normalized to the interval [0, 1] by dividing each vector by the length of the originating RNA sequence.

By utilizing a supervised model trained on the astrovirus sequences with known taxonomic labels, we were able to take advantage of established knowledge about this virus family. The supervised classification algorithms tested in this study are 10-Nearest Neighbours (Altman, 1992), Nearest Centroid Mean (Tibshirani et al., 2002), Nearest Centroid Median (Tibshirani et al., 2002), Logistic Regression (McCullagh and Nelder, 1989), Linear Support Vector Machines (SVM) (Cristianini and Shawe-Taylor, 2000), SVM with quadratic polynomial kernel (Quadratic SVM) (Cristianini and Shawe-Taylor, 2000), SVM with cubic polynomial kernel (Cubic SVM) (Cristianini and Shawe-Taylor, 2000), SVM with stochastic gradient descent learning and linear kernel function (SGD) (Cristianini and Shawe-Taylor, 2000), Decision Tree (Breiman et al., 1984), Random Forest (Breiman, 2001), AdaBoost (Freund and Schapire, 1997), Gaussian Naive Bayes (Chan et al., 1982), Linear Discriminant Analysis (LDA) (Hastie et al., 2009), Quadratic Discriminant Analysis (QDA) (Hastie et al., 2009), and Multilayer Perceptron (MLP) (Hinton, 1990; Kingma and Ba, 2015). Python library scikit-learn’s implementations of the fifteen aforementioned classifiers (Pedregosa et al., 2011) were used. Supplementary Material S3 (Performance Results of 3PCM Using Different Classification/Clustering Algorithms) list the experimental results of these fifteen candidate algorithms used in Prong 1 of 3PCM.

Only astrovirus RNA sequences in Dataset 2 with existing labels (described in Table 2) were used for training and testing for our initial experiment in order to select the most effective supervised classification algorithm among sixteen candidates as well as to demonstrate the effectiveness of Prong 1. As the testing dataset consisted only of sequences with known taxonomic labels, we could determine classification accuracy by comparing the predicted labels with the true labels. In order to assess the accuracy of the classifiers, we used Stratified 10-Fold Cross-Validation (Refaeilzadeh et al., 2009; Pedregosa et al., 2011). Based on the results presented in Supplementary Material S3 (Performance Results of 3PCM Using Different Classification/Clustering Algorithms), most of the model’s predictions match the true label (fourteen of sixteen algorithms achieved accuracy greater than 90%). Quadratic SVM and Cubic SVM were the most accurate algorithms for classifying astrovirus whole genomes by achieving an accuracy of 99.56%. Consequently, Quadratic SVM was selected as the classification algorithm in Prong 1 for the remainder of this paper.

To further assess the performance of 3PCM’s Prong 1, we conducted a comparative analysis by benchmarking our outcomes against two leading alignment-free machine-learning genome classification methods suitable for viral classification: Machine Learning with Digital Signal Processing (ML-DSP) (Randhawa et al., 2019; Randhawa et al., 2020) and the Viral Genome Deep Classifier (VGDC) (Fabijańska and Grabowski, 2019). The performance comparison between these two methods and the proposed Prong 1 with Quadratic SVM is detailed in Table 4, based on experiments using Dataset 2 with 10-fold cross-validation. As seen in Table 4, Prong 1 achieves superior classification accuracy compared to both ML-DSP and VGDC by margins of 0.56% and 3.68%, respectively.

**TABLE 4:** Classification accuracy of 3PCM’s Prong 1 against two state-of-the-art alignment-free machine-learning viral genome classification methods (ML-DSP, VGDC) using 10-fold cross-validation technique.

Classifier	Classification accuracy (%)
Prong 1 (Quadratic SVM)	**99.56**
ML-DSP	99.00
VGDC	95.88

The values in this table are averages over 10 different validation datasets. Bold value in the table indicate the highest value of the evaluation metric (classification accuracy).

#### 2.2.4 Prong 2: unsupervised machine learning

Prong 2 of the proposed classification method is unsupervised clustering, which is agnostic to and independent of taxonomic labels and annotations. Taking into account the possibility that the current classification of viruses based solely on their host may be flawed or incomplete due to limited information, knowledge, or characterization, it was necessary to use an alternate approach that does not rely on current labels. The use of unsupervised clustering alongside Prong 1 (supervised learning) allowed for the flexible use of as yet unclassified and unannotated astrovirus genomes in the training phase. Approximately one-third of astrovirus sequences are as yet unclassified (308 out of 992) and cannot be used in supervised models as they lack “ground truth” taxonomic labels. The potential inclusion of these sequences in the clustering model allows for the examination of the hypothesis that astrovirus consists of more than two genera (*Mamastrovirus* and *Avastrovirus*), which was not possible in Prong 1.

In Prong 2, the same feature vectors as in Prong 1 (*k*-mer counts) were used. The performance of Prong 2 for different values of *k* in the range [1, 9] in terms of classification accuracy and time can be found in Supplementary Material S3 (Performance Results of 3PCM Using Different Classification/Clustering Algorithms). The value *k* = 6 was empirically found to achieve the best balance between accuracy and computational complexity for the datasets and computational experiments in this paper. Furthermore, to find the most suitable clustering algorithm, we calculated and normalized the feature vectors and then tested three different clustering algorithms, K-means++ (Arthur and Vassilvitskii, 2007), Gaussian Mixture Model (GMM) (Dempster et al., 1977), and Hierarchical Clustering (Bridges Jr, 1966). We used Python library scikit-learn’s implementations of the three candidate clustering algorithms (Pedregosa et al., 2011). These algorithms were chosen due to their effectiveness in RNA classification (Kraskov et al., 2005; Akhtar et al., 2007; Aleb and Labidi, 2015; Hoang et al., 2015; Bustamam et al., 2017; James et al., 2018; Mendizabal-Ruiz et al., 2018).

These three clustering algorithms were compared by calculating the silhouette coefficient (Rousseeuw, 1987) as an internal evaluation metric ranging from −1 to 1, with higher values indicative of better clustering performance. In addition, we calculated external evaluation metrics such as Normalized Mutual Information (NMI) (Strehl and Ghosh, 2002), Adjusted Rand Index (ARI) (Rand, 1971), and classification accuracy to further compare the five clustering algorithms. NMI values range from 0 to 1, with 1 indicating perfect agreement and 0 indicating no agreement between these two clusterings. ARI values range from −1 to 1, where a value of 1 indicates perfect agreement between predicted and true labels, a value of 0 indicates no agreement and negative values indicate disagreement. We calculated classification accuracy of the clustering algorithms by using the Hungarian algorithm (Kuhn, 1955) in a *post hoc* step. This algorithm identifies the optimal mapping between the numerical cluster labels obtained by the clustering algorithms and the true taxonomic cluster labels. In external evaluation metrics, the results of clustering are compared with some known ground truth or with a reference set of labels. Consequently, we focused on sequences that had already been established as belonging to the *Mamastrovirus* and *Avastrovirus* genera of the *Astroviridae* family (Dataset 2 described in Table 2) and used this information for calculating external evaluation metrics. Please refer to Supplementary Material S3 (Performance Results for 3PCM Testing of Multiple Accuracy Classifiers) to see implementation details and the performance results of the clustering of the three clustering algorithm candidates measured in terms of the aforementioned internal and external evaluation metrics.

**FIGURE 2:**
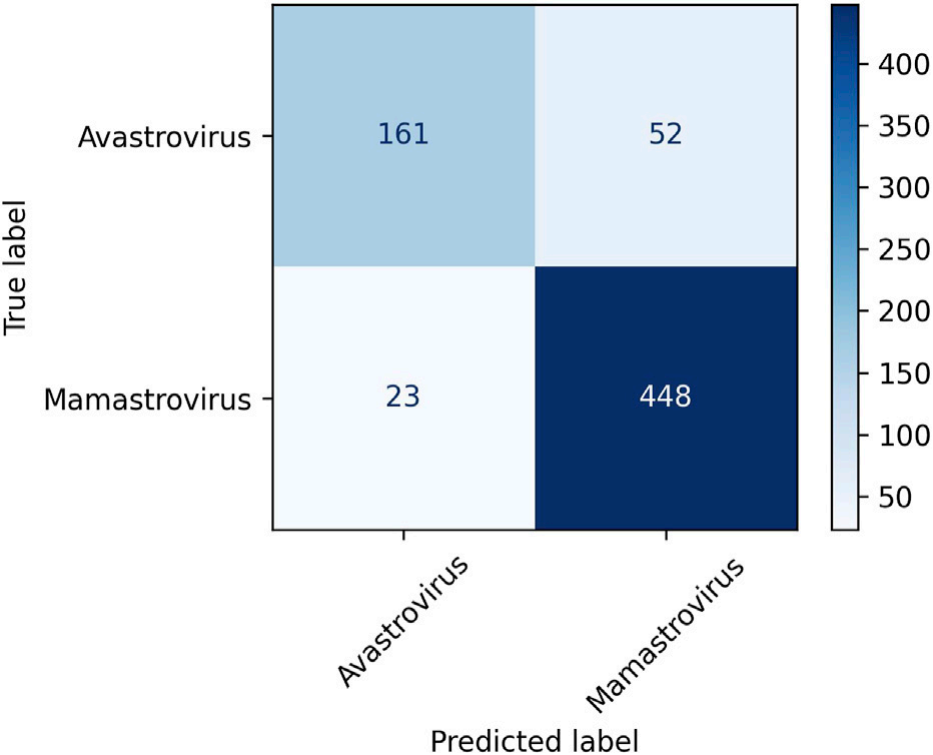
Confusion matrix for the clustering of Dataset 2, the astrovirus whole genomes with available taxonomic labels (dataset described in Table 2) into the *Avastrovirus* and *Mamastrovirus* genera using K-means++ algorithm in Prong 2 of 3PCM.

Among the three clustering algorithms, K-means++ performs the best in terms of all four evaluation metrics. K-means++ succeeded to achieve a classification accuracy of 88.16% NMI of 0.45, ARI of 0.58, and silhouette coefficient of 0.08. Consequently, K-means++ was selected as the clustering algorithm in Prong 2 for the remainder of this study. Figure 2 displays the confusion matrix obtained from clustering Dataset 2 using the K-means++ algorithm in Prong 2 of 3PCM. According to the figure, 448 out of 471 *Mamastroviruses* and 161 out of 213 *Avastroviruses* were clustered correctly. Major misclustering occurred for 52 *Avastroviruses* that were grouped with the majority of *Mamastroviruses*. It is possible that this is the result of an over-representation of *Mamastrovirus* over *Avastrovirus* in the dataset. Another possible explanation for this misclustering is the possibility of the existence of additional genera or subgenera inside the family *Astroviridae* which will be investigated in the Results section.

To further evaluate the performance of 3PCM’s Prong 2, we conducted a comparative analysis by contrasting our results with three state-of-the-art alignment-free machine-learning genome clustering methods suitable for viral sequences: Deep Learning for Unsupervised Classification of DNA Sequences (DeLUCS) (Millán Arias et al., 2022), its enhanced and interactive version (*i*DeLUCS) (Millan Arias et al., 2023), and MeShClust v3.0 (Girgis, 2022). DeLUCS and *i*DeLUCS rely on deep learning to uncover patterns (genomic signatures) within raw, unlabeled primary RNA/DNA sequence data, while MeShClust employs a mean-shift algorithm on pairwise alignment-free identity scores. The performance comparison of these three clustering methods and the proposed Prong 2 with K-means++ is presented in Table 5, based on experiments utilizing Dataset 2 for both the testing and the training phases.

**TABLE 5:** Performance of 3PCM’s Prong 2 against three state-of-the-art alignment-free machine-learning viral genome clustering methods (DeLUCS, *i*DeLUCS, and MeShClust v3.0) for clustering DNA sequences of the family *Astroviridae*, with available taxonomic labels at the genera level (Dataset 2).

Clustering algorithm	Classification accuracy (%)	NMI [-1,1]	ARI [-1,1]	Silhouette coefficient [0,1]
Prong 2 (K-means++)	**88.16**	**0.45**	**0.58**	**0.08**
DeLUCS	66.40	0.17	0.11	**0.08**
*i*DeLUCS	66.01	0.11	0.10	0.04
MeShClust v3.0	65.20	0.03	−0.03	0.005

Classification accuracy, NMI [−1, 1], ARI [−1, 1], and silhouette coefficient [0, 1] were employed as evaluation metrics. Higher values indicate better performance for all evaluation metrics. Bold values in the table indicate the highest value of the evaluation metrics (classification accuracy, NMI, ARI, and silhouette coefficient).

The computational experiments involving DeLUCS and *i*DeLUCS showed that increasing the mutation rate to *p*_*ts*_ = 10^–3^ and *p*_*tv*_ = 0.5 × 10^−3^ (rather than the default values *p*_*ts*_ = 10^–4^ and *p*_*tv*_ = 0.5 × 10^−4^) and using 9 mimic sequences (rather than the default value of 3) increased the accuracy of astrovirus genome clustering. Due to the variability in the results of DeLUCS, the results reported in Table 5 are the average values over 10 different runs. In contrast to the other methods, MeShClust does not allow for the pre-setting of the number of clusters due to its density-based nature. As a result, multiple values were examined for the identity score threshold within the range of [0, 1], and the value of 0.4005 was selected as it was the one resulting in two clusters. Overall, as shown in Table 5, Prong 2 outperforms DeLUCS, *i*DeLUCS, and MeShClust in terms of both internal and external evaluation metrics, with a classification accuracy that is 21.76%–22.96% higher than the other three methods.

The proposed methodology was further tested by collecting 1,450 genomes of the closest viral family to astrovirus, namely, potyvirus (*Potyviridae*). Using potyvirus genome sequences, a dataset consisting of all available astrovirus and potyvirus genomes was created and classification/clustering of these two viral families was tested using 3PCM. The exclusion of recombinants from this dataset was not performed due to the rarity of interspecific recombination within potyvirus. Additionally, no recombination is anticipated between potyvirus and astrovirus (Gibbs et al., 2020). Prong 1 (supervised) and Prong 2 (unsupervised) achieved accuracies of 99.8% and 93.47%, respectively. The accuracies achieved provide compelling evidence of the effectiveness of 3PCM in the classification/clustering of viral genomes at different taxonomic levels. Details of these computational experiments can be found in Supplementary Material S4 (Astrovirus Near-Neighbour Analysis: Potyvirus).

Lastly, 3PCM was used at a lower taxonomic level, for the classification/clustering of *Avastrovirus* and *Mamastrovirus* genera into different subgroups based on their host species. This test was augmented by a principal component analysis (PCA) of the *k*-mer composition of the astrovirus genomes, for *k* = 6.

### 2.3 Computational setup

The laptop used for data collection and recombinants analysis was a Lenovo L-series ThinkPad with an intel core i5 processor and 32 GB ram. Datasets consisting of *Astroviridae* and Patatavirales in this study were retrieved from the National Center for Biotechnology Information (NBCI). Sequences were downloaded using the application Geneious Prime 2022.1 https://www.geneious.com/ via the NCBI nucleotide database.

We empirically selected the hyperparameters of different classification/clustering algorithms that yielded the best performance during the training procedure. Both Prong 1 and Prong 2 of 3PCM are implemented in Python 3.10 and the source code, as well as all the datasets used in this paper, are publicly available in the GitHub repository https://github.com/fatemehalipour/3PCM. All of the tests were performed on Google Colab Pro environment [2 x Intel(R) Xeon(R) CPU @ 2.20 GHz, 32 GB RAM] with NVIDIA A100 GPU.

## 3 Results

In this section, we showcase the outcomes achieved through the pipeline explained in the preceding section, Materials and Methods. First, the results of applying recombinant elimination to astrovirus sequences will be discussed. Following that, we will present the results of the novel classification method applied to as yet unclassified astroviruses. Lastly, the existence of subgenera within *Mamastrovirus* and *Avastrovirus* genera, as suggested by our observations, will be explored.

### 3.1 Identification and elimination of recombinant sequences

The optional component to eliminate recombinants was employed to examine Dataset 1, the primary dataset used in this study. As a result, 54 sequences (5.4% of the dataset) involved in interspecific recombination, associated with 34 recombination events, were identified. Notably, out of these 54 recombinations, 7 were intergeneric. Although the taxonomic classification task performed in this study is at the genus level, the presence of a negligible number (7) of intergeneric recombinations (which would yield noticeable variations in evaluation metrics) led to the decision to eliminate all 54 recombinants. For more detailed information, please refer to Supplementary Material S2 (Identification and Analysis of Candidate Recombinant Astrovirus Genome Sequences).

### 3.2 Classification of unclassified astroviruses with mammal and avian hosts

Using 3PCM, we attempted to predict taxonomic classification for as yet unclassified astrovirus sequences. The predictive models of 3PCM’s Prong 1 (supervised) and Prong 2 (unsupervised) were trained on sequences with known taxonomic labels (*Mamastrovirus* or *Avastrovirus*), and later used to predict the genus of as yet unclassified astrovirus sequences. In Prong 3, the class level host labels of the input as yet unclassified astrovirus sequences were considered. In cases where all three labels agree, those labels were proposed as genus labels for the respective as yet unclassified astrovirus genomes. When the taxonomic labels predicted by Prong 1 and Prong 2 agree, but they differ from the host label found by Prong 3, they are considered tentative and subject to further investigation.

For this analysis, only sequences with mammalian or avian hosts were investigated, since the two supervised and unsupervised predictive models were trained only on *Mamastrovirus* and *Avastrovirus* genomes. The sequences obtained from 11 other hosts were discarded, resulting in Dataset 3 comprising 229 astroviruses with mammalian or avian hosts, as the testing dataset (Table 3). Details of the distribution of hosts for the 308 as yet unclassified astrovirus RNA sequences can be found in Supplementary Material S1 (Analysis of Astroviruses of Unknown Genus Label).

**FIGURE 3:**
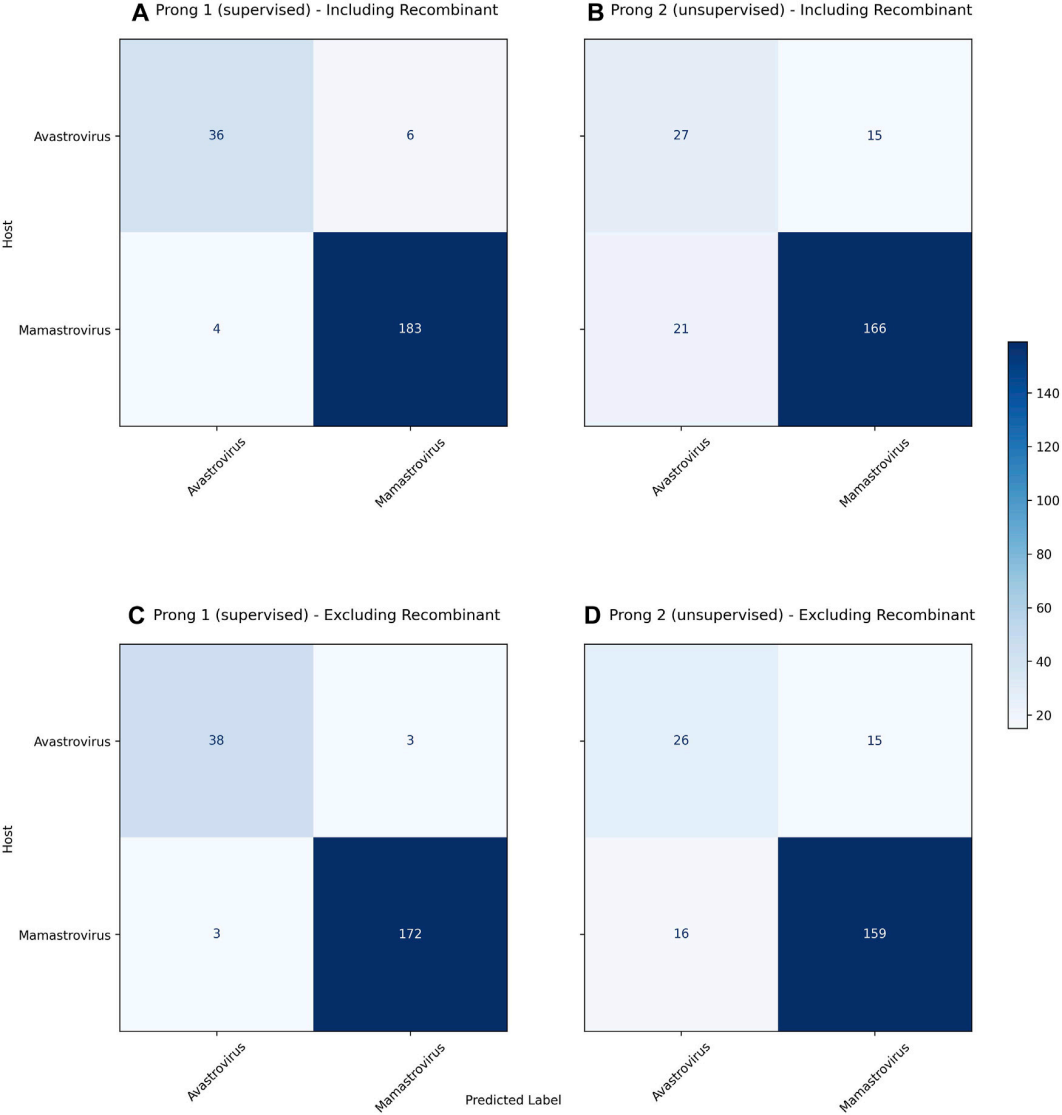
**(A)**: Confusion matrix for the classification of as yet unclassified astroviruses including recombinants using Prong 1 (supervised). **(B)**: Confusion matrix for clustering as yet unclassified astroviruses including recombinants using Prong 2 (unsupervised). **(C)**: Confusion matrix for the classification of as yet unclassified astroviruses excluding recombinants using Prong 1 (supervised). **(D)**: Confusion matrix for clustering as yet unclassified astroviruses excluding recombinants using Prong 2 (unsupervised). Prong 3, the host labels, was considered the ground truth for both confusion matrices.

Figure 3 displays the confusion matrices resulting from the use of Prong 1 (supervised) and Prong 2 (unsupervised) for the classification/clustering datasets including (top), respectively excluding (bottom) recombinant sequences. Note that, out of 54 identified recombinant sequences, 41 were present in the training set (Dataset 2), while the remaining 13 belong to the testing set (Dataset 3). Table 6 summarizes the results of the evaluation metrics for both Prong 1 (classification accuracy) and Prong 2 (clustering accuracy, NMI, ARI, Silhouette Coefficient). As seen from Figure 3 and Table 6, the accuracy of both Prong 1 and Prong 2 increased slightly when the recombinant sequences were removed from the training and test sets (by 1.59% in the case of supervised learning, and by 1.48% in the case of unsupervised clustering). This observation indicates the potential negative impact that recombination events can have on machine learning-based classification/clustering approaches. While the impact may not be highly significant in this analysis, it is crucial to recognize that this may not hold true in all cases. The effect of this elimination process will correlate with the extent to which recombination contributes to noise in the classification. This, in turn, varies based on virus biology, frequency within the dataset, and the taxonomic level of classification.

**TABLE 6:** Evaluation metrics of Prong 1 and Prong 2 applied to the classification/clustering unclassified astroviruses into *Mamastroviruses* and *Avastroviruses* when using datasets that include, respectively exclude recombinant sequences.

Method	Recombinants	Classification/Clustering accuracy (%)	NMI [−1, 1]	ARI [−1, 1]	Silhouette coefficient [0, 1]
Supervised (Quadratic SVM)	Included	95.63	N/A	N/A	N/A
Supervised (Quadratic SVM)	Excluded	**97.22**	N/A	N/A	N/A
Unsupervised (K-means++)	Included	84.15	0.22	0.39	**0.06**
Unsupervised (K-means++)	Excluded	**85.63**	**0.25**	**0.43**	**0.06**

Prong 3 host labels are considered the ground truth for calculating the external evaluation metrics [Normalized Mutual Information (NMI), Adjusted Rand Score (ARI), and Classification/Clustering Accuracy]. Higher values indicate better performance for all evaluation metrics. Values within the brackets indicate the range of each evaluation metric. Bold values in the table indicate the highest value of the evaluation metrics (classification accuracy, NMI, ARI, silhouette coefficient) in both supervised and unsupervised methods.

As seen in Table 7, when both the training and test datasets include recombinant sequences, all three prongs agree on 83.41% (191 out of 229) of the sequences in the testing dataset. For an additional 3% (8 out of 229) of the sequences, the Prong 1 and Prong 2 predictions agree, but differ from the Prong 3 prediction. When recombinant sequences are excluded from both the training and testing datasets, all three prongs agree on predictions for 85.65% (185 out of 216) of the sequences in the testing dataset. Similarly, for an additional 3% (6 out of 216) of the sequences, Prong 1 and Prong 2 agree in their predictions, while Prong 3 disagrees. The NCBI accession IDs of the as yet unclassified 191 + 8 astrovirus sequences when including recombinants and the as yet unclassified 185 + 6 astrovirus sequences when excluding recombinants, together with the taxonomic labels (at the genus level) predicted by 3PCM can be found in Supplementary Material S5 (Proposed Classification for as yet Unclassified Astroviruses). The group of 185 + 6 sequences, where 3PCM led to a genus level classification when excluding recombinants, is a subset of 191 + 8 sequences for which a classification was proposed using 3PCM when including recombinants, with the exception of one sequence with accession ID *MT*138006, for which the classification prediction was only generated when recombinants were excluded from the analysis.

**TABLE 7:** Number of as yet unclassified viral genomes for which two or all three, of the prongs, agree in their genus label prediction.

Including recombinants				
Prong 1 (supervised)	✓	✓	✓	✗
Prong 2 (unsupervised)	✓	✓	✗	✓
Prong 3 (host)	✓	✗	✓	✓
# Viral Genomes Agreed	**191 (229)**	**8 (229)**	28 (229)	2 (229)
Excluding recombinants				
Prong 1 (supervised)	✓	✓	✓	✗
Prong 2 (unsupervised)	✓	✓	✗	✓
Prong 3 (host)	✓	✗	✓	✓
# Viral Genomes Agreed	**185 (216)**	**6 (216)**	25 (216)	0 (216)

A checkmark indicates agreement between the prongs. For example, the first column represents the case when agreement of all three prongs occurs, the second column represents the case when Prong 1 and Prong 2 but not Prong 3 agree, etc. The numbers in parentheses represent the total size of the test dataset. Bold values in the table indicate the number of unclassified viral genomes for which Prong 1 and Prong 2 agreed in their genus label prediction.

Although both sets of results (with and without recombinants) are meaningful, we selected as the primary result of this paper the 191 + 8 genus label predictions obtained from the analysis that includes recombinants. This decision was mainly influenced by the fact that the majority of the recombination events observed are intrageneric, and are thus not significant at genus level classification/clustering.

For the 191 sequences for which all three prongs agree, 26 are predicted to belong to genus *Avastrovirus*, and the remainder 165 are predicted to belong to genus *Mamastrovirus.* For these 191 sequences, the proposed genus labels are more certain than for the 8 sequences for which Prong 3’s host label disagreed with Prong 1 and Prong 2’s predictions. For the latter, three out of eight sequences were classified as *Avastrovirus* by Prong 1 and Prong 2 despite being obtained from mammals. These three sequences are *JN*420353 [a California sea lion astrovirus (Li et al., 2011)], *MH*933754 [a human astrovirus (Yinda et al., 2019)], and *NC*_035758 [a human astrovirus (Orf et al., 2023)]. Regarding the remaining five sequences, obtained from an avian host, the first two prongs predicted that they belonged to the *Mamastrovirus* genus. The five sequences are: *KP*663426 (Pankovics et al., 2015), *MT*138010 (Shan et al., 2022), *NC*_027426 (Orf et al., 2023), *ON*304005 (French et al., 2022), *MK*096773 (Fernández-Correa et al., 2019). Further investigation is needed in order to determine the origin, and the spectrum of natural host species, of these eight sequences.

The genomes of as yet unclassified astroviruses with hosts other than mammals and avians were also examined, to determine whether they can be classified as belonging to the genera, *Mamastrovirus* or *Avastrovirus* or to detect whether this family of viruses has more than two genera. Prong 1 (supervised) was not applicable to this problem, due to the absence of ground truth labels in the training set. The clustering results obtained by using Prong 2 (unsupervised) showed no clear separation among the unclassified astroviruses with non-mammalian/non-avian hosts, nor was there any clear separation found between these genomes and *Avastroviruses* or *Mamastroviruses*. This could potentially be due to a lack of availability of sufficiently many genomes with non-mammalian and non-avian hosts, which can negatively affect the efficacy of machine learning methods. Details of these computational experiments can be found in Supplementary Material S1 (Analysis of Astroviruses of Unknown Genus Label).

### 3.3 A closer look at genera *Mamastrovirus* and *Avastrovirus*

**FIGURE 4:**
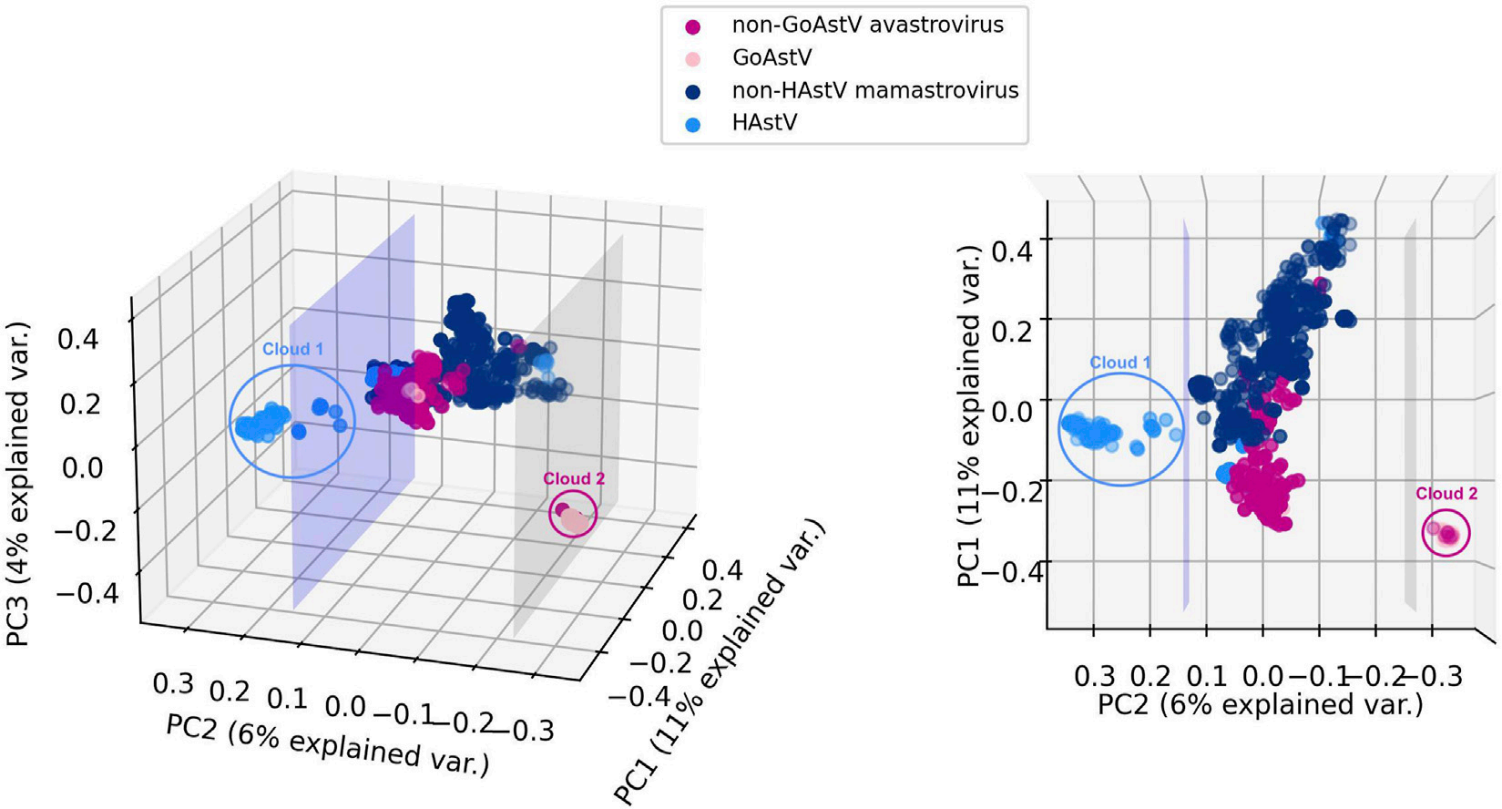
Two views of 3D PCA data visualizations of *Mamastrovirus* and *Avastrovirus* sequences *k*-mer frequencies: astrovirus sequences in Dataset 2 (known genus labels), together with the 191 astrovirus genomes with genus labels predicted by 3PCM. For comparison purposes, *HAstV* and *GoAstV* are highlighted with different colors compared to the rest of *Mamastroviruses* (*non-HAstV* *Mamastroviruses*) respectively the rest of the *Avastroviruses* (*non-GoAstV* *Avastroviruses*). The lavender plane illustrates the separation between two possible subgenera of *Mamastrovirus.* The grey plane illustrates the separation between two possible subgenera of *Avastrovirus*. This visualization is based on the first three principal components of 6-mer counts for the entire genome. In this figure, Clouds 1 and 2 represent possible subgenera of *HAstV* (Cloud 1) within genus* Mamastrovirus*, and *GoAstV* (Cloud 2) within genus *Avastrovirus*.

Given the mounting evidence for different serotypes, clades, and genotypes associated with unique cross-species transmissions, different rates of evolution and intraspecific recombination for human astroviruses (HAstV) (Bosch et al., 2014; Donato and Vijaykrishna, 2017; Hargest et al., 2021; Perez et al., 2023) and goose astroviruses (GoAstV) (Fei et al., 2022; Zhu and Sun, 2022), we further investigated the sequences belonging to these two subgroups. Figure 4 displays a visualization of the 6-mer counts of the genomes in Dataset 2 (*Mamastrovirus* and *Avastrovirus* genomes with established labels), together with the 191 genomes with genus labels predicted by 3PCM with the use of principal component analysis (PCA). For visualization purposes, the first three principal components of the 6-mer counts for each genome are utilized, preserving ∼21% of the explained variance.

The first step in this analysis was to extract information about the host species. The available 636 *Mamastrovirus* sequences with a host label at the species level were obtained from 69 different species. Of these, 362 sequences were obtained from the four most representative hosts, 105 from *Sus scrofa*, 44 from *Sus domesticus*, 153 from *Homo sapiens*, and 60 from *Bos taurus*.

As seen in Figure 4, a separating plane exists that separates Cloud 1 (111 *Mamastrovirus* genomes) from the rest of the *Mamastrovirus* genomes. A closer examination reveals that, while not all 163 *HAstV* (human host) sequences are in Cloud 1, all 111 sequences in Cloud 1 are *HAstV* sequences. This suggests that a *HAstV* subgenus exists within the genus *Mamastrovirus.* A comparison between Cloud 1 and the human *Mamastroviruses* sequences analyzed in Perez et al. (2023) reveals that all the 91 MAstV-Sp7G3 human astrovirus sequences included in our analysis (not collected from sewage, not collected from an unknown host, etc.) are located in Cloud 1. Moreover, all 13 MAstV-Sp6G2 sequences and all 18 MAstV-Sp6G7 sequences analyzed in Perez et al. (2023) are separated from Cloud 1 and located in the main cloud (the sequences located between the two separating planes). This suggests a correspondence between the Cloud 1 sequences and MAstV-Sp7G3 sequences. The accession IDs of sequences in Cloud 1 can be found in Supplementary Material S1 (Analysis of Astroviruses of Unknown Genus Label).

To further examine this hypothesis, 3PCM was applied to the genome sequences of the *Mamastrovirus* genus (the aforementioned 636 *Mamastrovirus* genome sequences), with the labels being *HAstV* and *Non-HAstV* *Mamastrovirus* depending on the sequences’ host species. The accuracies of applying Prong 1 (supervised) and Prong 2 (unsupervised) to this dataset, computed using Prong 3 (host labels) as the ground truth, are shown in Table 8. The high classification accuracy of Prong 1 (99.36%) and unsupervised clustering accuracy of Prong 2 (80.88%), provide additional evidence supporting the hypothesis of the existence of a *HAstV* subgenus of the genus *Mamastrovirus.* Using the available data, no separation is evident for the other hosts of *Mamastroviruses*.

**TABLE 8:** Evaluation metrics of Prong 1 and Prong 2 applied to the classification/clustering of *Mamastrovirus* sequences into *HAstV* and *Non-HAstV* *Mamastrovirus.*

Method	Classification/Clustering accuracy (%)	NMI [−1, 1]	ARI [−1, 1]	Silhouette coefficient [0, 1]
Supervised (Quadratic SVM)	99.36	N/A	N/A	N/A
Unsupervised (K-means++)	80.88	0.34	0.37	0.07

Prong 3 host labels are considered the ground truth for calculating the external evaluation metrics [Normalized Mutual Information (NMI), Adjusted Rand Score (ARI), and Classification/Clustering Accuracy]. Higher values indicate better performance for all evaluation metrics. Values within the brackets indicate the range of each evaluation metric.

When investigating the host species of genus *Avastrovirus*, we analyzed the 239 *Avastroviruses* with available host species labels, obtained from 28 different species. Of these, 135 sequences were obtained from the two most representative hosts, 64 from Goose and 71 from Chicken (*Gallus gallus*).

As seen in Figure 4, a separating plane exists that separates Cloud 2 (66 *Avastrovirus* genomes) from the rest of the *Avastrovirus* genomes. Further investigation of Cloud 2 revealed that 59 of its sequences have a *Goose* host (GoAstV sequences), amounting to 92% (59 out of 64) of the available *GoAstV* sequences. This suggests that a *GoAstV* subgenus may exist within the genus *Avastrovirus*. A comparison between the sequences in Cloud 2 and two established genotypes of *GoAstV* (see Zhu and Sun, 2022) reveals that all 51 *GoAstV*-2 (G2) goose astrovirus sequences analyzed in Zhu and Sun (2022) are located in Cloud 2, and all 5 *GoAstV*-1 (G1) sequences analyzed in Zhu and Sun (2022) are separated from Cloud 2 and located in the main cloud (the sequences located between the two separating planes). This suggests that the observed separation of the Cloud 2 *GoAstV* sequences from the rest corresponds to the aforementioned two genotypes of the *GoAstV* virus analyzed in Zhu and Sun (2022). The accession IDs of sequences in Cloud 2 can be found in Supplementary Material S1 (Analysis of Astroviruses of Unknown Genus Label).

To further examine this hypothesis, 3PCM was applied to the genome sequences in this dataset, with the labels being *GoAstV* and *Non-GoAstV* *Avastrovirus* depending on the sequence’s host species. The classification/clustering accuracies of applying Prong 1 (supervised) and Prong 2 (unsupervised) to this dataset, computed using Prong 3 (host labels) as the ground truth, are shown in Table 9. The high classification accuracy of Prong 1 (94.96%), and unsupervised clustering accuracy of Prong 2 (94.98%), provide additional evidence supporting the hypothesis of the existence of a *GoAstV* subgenus of the genus *Avastrovirus*. According to the available data, no separation is apparent for the other hosts of *Avastroviruses*.

**TABLE 9:** Evaluation metrics of Prong 1 and Prong 2 applied to the classification/clustering of *Avastrovirus* sequences into *GoAstV* and *Non-GoAstV* *Avastrovirus*.

Method	Classification/Clustering accuracy (%)	NMI [−1, 1]	ARI [−1, 1]	Silhouette coefficient [0, 1]
Supervised (Quadratic SVM)	94.96	N/A	N/A	N/A
Unsupervised (K-means++)	94.98	0.67	0.80	0.21

Prong 3 host labels are considered the ground truth for calculating the external evaluation metrics [Normalized Mutual Information (NMI), Adjusted Rand Score (ARI), and Classification/Clustering Accuracy]. Higher values indicate better performance for all evaluation metrics. Values within the brackets indicate the range of each evaluation metric.

## 4 Discussion

We introduce the Three-Pronged Classification Method (3PCM), a novel approach that integrates both supervised and unsupervised machine learning paradigms, along with information about the originating species, to classify emerging astroviruses. The main objective of this study was to suggest a classification system for 229 as yet unclassified astrovirus sequences acquired from avian and mammalian hosts. This approach was taken due to the limited number of available sequences and the lack of definitive information on other hosts. Out of the 229 as yet unclassified sequences, the three-pronged classification yielded consistent predictions for 191 of them, indicating a very high level of reliability for the proposed classification. Furthermore, among the as yet unclassified sequences, For 8 additional sequences, the computational predictions of Prong 2 and Prong 2 coincided, but were different from the host information obtained by Prong 3. In light of numerous supporting evidence regarding the possibility of cross-species infection Pankovics et al. (2015), the classification proposed by Prong 1 and Prong 2 takes precedence over Prong 3. With the investigation in literature, we were able to validate the taxonomic classification labels proposed by Prong 1 and Prong 2, confirming the existence of cross-species infection in both *Mamastroviruses* and *Avastroviruses* in these sequences.

3PCM’s versatility lies in its ability to employ each of the three prongs independently or in combination, providing a highly adaptable classification method suitable for various taxonomy tasks. The hypothesis of the existence of additional *Avastrovirus* and *Mamastrovirus* genera associated with astroviruses from Reptiles, Amphibians, and Actinopterigii hosts was explored using Prong 2 and Prong 3, which are not reliant on sequence labels. The results showed no clear separation between these three groups and *Mamastrovirus* and *Avastroviruses*, but the reason for this could be the limited availability of sequences. For a more comprehensive and accurate analysis, the identification of more Astrovirus sequences from hosts beyond avians and mammalians is necessary.

Elimination of recombinant sequences is an optional step in the 3PCM pipeline. We identified 54 instances of interspecies recombination in Dataset 1, seven of which were intergeneric. The classification/clustering accuracy of Prongs 1 and 2 increased by 1%–2% after the 54 recombinant candidates were removed. Since this study was concerned with genus level classification, of all recombination events only intergeneric recombinations could have an influence on the classification accuracy. As the number of intergeneric recombination events detected in this dataset was low (7 out of 992), the impact of the removal of recombinant sequences on classification accuracies was expected to be negligible. Nonetheless, the option to include this step in the pipeline is essential, as its impact on classification accuracy may vary, depending on the level of taxonomic classification and the frequency and nature of genetic recombination in the virus genomes being classified. The expert user can decide whether or not to include this option, based on the specific virus biology (including the propensity for recombination and whether it is intra or intergeneric), the frequency of such recombinations in the dataset, and the level of classification (e.g., genus or species).

Since the recognition of *Astroviridae* as a family in 1993, this group of viruses infected over 140 hosts across the globe and is the second largest cause of gastroenteritis in humans. Due to the rapid expansion of infected hosts, frequent inter-species transmission, and genetic recombination, traditional classification based solely on a host may be insufficient. This paper presented 3PCM, a novel machine-learning classification method utilizing both virus-host and whole-genome composition. To enhance the effectiveness of 3PCM, an optional component was added to the pipeline that is responsible for eliminating recombinant sequences. Following the classification of the as yet unclassified astroviruses using Prong 1 (supervised classification) and Prong 2 (unsupervised clustering), the NCBI host labels were used as possible ground truth to classify/cluster astrovirus whole genome sequences, with an accuracy of 95.63%, and 84.15%, respectively. From this classification method, we propose 26 avian-host-derived sequences and 165 mammalian host-derived sequences be added to *Avastrovirus* and *Mamastrovirus* genera, respectively. A taxonomic classification was also proposed for eight additional as yet unclassified astrovirus sequences, which are not aligned with the host species of the sequences and may be capable of transmitting across species. As anticipated, the need for a rapid and multipronged approach for astrovirus classification continues to grow (the number of unclassified genome sequences grew from 308 in July 2022 to 429 in September 2023). The 3PCM pipeline is available for ongoing classification of newly added sequences and its power increases with the informative increase in the modeling.

Furthermore, 3PCM was used to investigate the hypothesis of the existence of subgenera *GoAstV* and *HAstV* within *Avastrovirus* and *Mamastrovirus* respectively. Using 3PCM for classification/clustering of the genus *Mamastrovirus* into *HAstV* and *Non-HAstV Mamastrovirus*, accuracies of 99.36%, and 80.88% for Prong 1 and Prong 2 were achieved. Furthermore, the accuracy of 94.96% and 94.98% were achieved when Prong 1 and Prong 2 were used for classification/clustering of the genus *Avastrovirus* into *GoAstV* and *Non-GoAstV*, respectively. The results of these two experiments were further verified by an investigation of the difference in the genome composition of the subgroups. As a result, we propose that each of these subgroups is a distinct sub-genus.

## Data Availability

The original contributions presented in the study are included in the article/Supplementary Material, further inquiries can be directed to the corresponding author. The datasets collected and analyzed for this study can be found in the GitHub repository https://github.com/fatemehalipour/3PCM.

## Supplementary material

SUPPLEMENTARY DATA SHEET S1 Supplementary Material S1: Analysis of Astroviruses of Unknown Genus Label.

SUPPLEMENTARY DATA SHEET S2 Supplementary Material S2: Identification and Analysis of Candidate Recombinant Astrovirus Genome Sequences.

SUPPLEMENTARY DATA SHEET S3 Supplementary Material S3: Performance Results of 3PCM Using Different Classification/Clustering Algorithms.

SUPPLEMENTARY DATA SHEET S4 Supplementary Material S4: Astrovirus Near-Neighbour Analysis (Potyvirus).

SUPPLEMENTARY DATA SHEET S5 Supplementary Material S5: Proposed Classification for as yet Unclassified Astroviruses.
