# Supplementary material for: Leveraging machine learning for taxonomic classification of emerging astroviruses
Source: Front Mol Biosci. 2024 Jan 11;10:1305506. doi: 10.3389/fmolb.2023.1305506 (PMC10808839; doi:10.3389/fmolb.2023.1305506)
Supplement: Supplementary file 1 [file DataSheet2.PDF]

Supplementary Material 2: Identification and Analysis of Candidate Recombinant Astrovirus Genome Sequences

In part, the genetic diversity associated with astroviruses is associated with genetic recombination. The nature of the multiplicity of viral infection possible for astroviruses enables recombination between viruses of different taxa thereby potentially increasing genetic diversity significantly. The resultant recombinant mosaic genome may present challenges to alignment-free (e.g. k-mer based) approaches based solely on the primary genome sequence. To assess the impact of recombinant genomes upon machine learning based classification approaches, the virus dataset was analyzed for identification of candidate recombinants and testing was carried out for the genome datasets with and without the candidate intergeneric recombinants.

The multifasta file containing aligned astrovirus sequences was uploaded to the Recombination Detection Program $RDP4$. To identify potential recombination events, tools RDP, $GENECONV$, $BOOTSCAN$, $MaxChi$, $SiScan$, $CHIMEARA$, and $TOPAL$ were run to examine all possible sequence triplets and identify a recombinant sequence, major parent and minor parent. When one or more programs identified recombination signals, $RDP4$ utilized BURT as a hidden Markov model to identify where recombination breakpoint positions resided in the genome. $Phylpro$, $EEEP$, and $VisRD$ techniques were then leveraged to propose a recombinant sequence. A CSV file was exported with potential recombination events and tool results for further analysis. A region count matrix was calculated using $RDP4$ to generate a heatmap of detected Astroviridae recombinant sequence loci.

Detection of potential triplets of a recombinant, major parent, and minor parent was carried out to identify candidate recombination events. Three hundred eighty-two recombination events were identified by at least one of the seven tools employed. We focused on events identified by at least two tools, narrowing potential recombination events to 36. Hosts of origin are visualized in Figure 1. The genus Sus was the host involved in the most recombination events, followed by the genus Homo. In total when considering major and minor parents, 162 sequences were identified to be involved in recombination events, out of which 54 unique sequences were proposed to be recombinant sequences, equivalent to 5.4% of the population of 992 sequences analyzed. Table S1 represents the accession IDs of the 54 sequences that were identified as potential recombinants.

A recombination region count matrix was created to understand further where recombination was occurring in the genome. Each recombination event proposed was mapped based on the recombinant's estimated breakpoint positions. The colours represent the frequency at which recombination events are hypothesized to have occurred at a given site. Most recombination events appear to have occurred over the 5 kb to 7 kb region of the genome, approximately the location of ORF2.

As part of our classification pipeline, we removed the recombinant sequences from the data before training supervised and unsupervised models. Recombination can make phylogenetic analysis challenging, as it can connect distant lineages and confound a vertical pattern of evolution. By leveraging recombination analysis, we identified 36 potential recombination events within the Family Astroviridae. To our knowledge, this is the most identified in a singular test, and most of these candidate events are novel. One hundred sixty two unique sequences were potentially involved in recombination events considering major and minor parents in addition to the recombinants sequences, resulting in a high recombination rate of up to 16.4%. Of 192

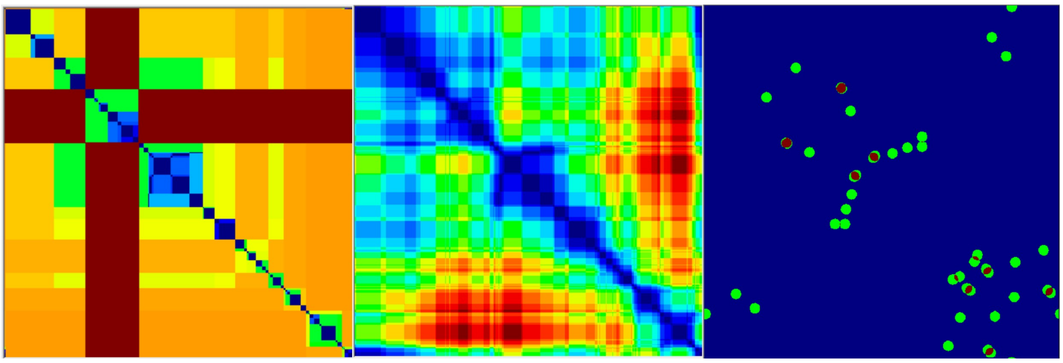

Figure S1. Recombination matrix (Left), region count matrix (Center), and breakpoint matrix (Right) for 162 astroviruses. Location in the genome progresses from the top left of each image to the top right and from the top left to the bottom left. Heatmap colours are associated with the number of recombination events at a site in the genome (red being the most and blue the least).

Table S1. Accession IDs of the identified recombinant sequences.

MK404646	DQ028633	MH933753	MK059955	MK378474	JX556690	MK378521
MT549855	KX033447	FJ755404	NC_026814	KP404149	KX756441	KF374704
KY765684	MW373720	MW784093	MZ325583	MZ325582	Y15937	NC_002469
NC_033792	KY024237	KX599353	KF039911	GU223905	MW588064	MG693176
MK671309	MK671308	MK671310	MN148428	MK987100	MK671312	ON304005
OM480542	MK378505	MW784090	NC_023629	HQ916316	KY214437	MZ357116
DQ344027	KJ020899	NC_019028	AB829252	JF742759	JX857870	NC_016155
MK404649	MK404647	KY940077	OM105048	KT946725	-	-

total recombination sequences, some of the 162 unique sequences being repeated, 92 recombinant viruses were identified in domestic or farm animals consisting of pigs, cows, sheep, cats, dogs, chickens, and goats. Moreover, viruses infecting humans were the second most common participant in recombination events, accentuating the population's increasing risk of zoonotic infection. However, a disproportionate number of domesticated animal and human hosts were sequenced, which limits generalization regarding patterns in localization of recombination more broadly. Results from a recombination region count matrix displayed almost all recombination events in the ORF2 region. Therefore, ORF2 volatility may contribute to the rapid expansion of astrovirus throughout a broad range of hosts. Astrovirus broad host range, transpecific infections, multiplicity of infection, and resultant opportunities for recombination have the potential to confound classification. Candidate recombinants can be identified and their inclusion and exclusion from a classification pipeline be assessed for likely impact on specific classification tests based on the amount and nature of recombination, and the candidate recombinants can be tested empirically for effects on classification.
