# Supplementary material for: Leveraging machine learning for taxonomic classification of emerging astroviruses
Source: Front Mol Biosci. 2024 Jan 11;10:1305506. doi: 10.3389/fmolb.2023.1305506 (PMC10808839; doi:10.3389/fmolb.2023.1305506)
Supplement: Supplementary file 2 [file DataSheet4.PDF]

# Supplementary Material 4: Astrovirus Near-Neighbour Analysis (Potyvirus)

As a test of the effectiveness of 3PCM, data on a near viral family to astrovirus, which is potyvirus (Potyviridae), the sole family of the Patatavirales order was collected. The Stellavirales order, which astrovirus belongs to, and the Patatavirales order are the only two orders categorized under the Stelpaviricetes class. Taxonomic classification of the Stelpaviricetes class is shown in Figure S1.

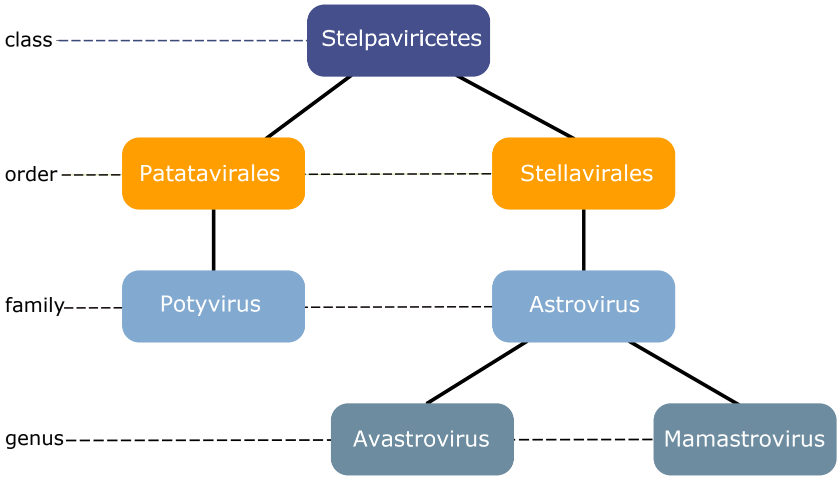


**Figure S1.** Taxonomic classification of class Stelpaviricetes.

A total of 1,450 sequences of the Family Potyviridae were downloaded from the NCBI database on August 30th, 2022. The sequences were filtered so that they are between 4 kbp and 10 kbp in length. This dataset comprises all 992 astrovirus sequences from Dataset 1 augmented with 1,450 potyvirus sequences and is described in Table S1.

**Table S1.** Description of the dataset comprising 2,422 genomes of viruses belonging to the Class Stelpaviricetes. Astrovirus and potyvirus are the only two families within this viral class.

| Genus | No. of sequences | Min. sequence length (bp) | Avg. sequence length (bp) | Max. sequence length (bp) |
|---|---|---|---|---|
| Astrovirus | 992 | 5,003 | 6,600 | 8,840 |
| Potyvirus | 1,450 | 4,005 | 8,676 | 9,585 |
| All/Average | 2,442 | 4,005 | 7,833 | 9,585 |

Table S2 summarizes the results of the evaluation metrics for both Prong 1 (classification accuracy) and Prong 2 (clustering accuracy, NMI, ARI, Silhouette Coefficient). The experiment of Prong 1 was performed by using 10-fold cross-validation, and the experiment of Prong 2 was conducted 20 times with varying initialization of the K-Means++ model. In Table S2, the values represent the average value of the twenty

runs. By computing all metrics used to evaluate classification/clustering performance, both Prong 1 and Prong 2 can separate potyviruses from astroviruses with high accuracy.

**Table S2.** Evaluation metrics of Prong 1 and Prong 2 applied to the classification/clustering of RNA sequences of the Stelpaviricetes class into family Astrovirus and family Potyvirus. Prong 3 host labels are considered the ground truth for calculating the external evaluation metrics (NMI, ARI, Classification/Clustering Accuracy). Higher values indicate better performance for all evaluation metrics.

| Method | Accuracy | NMI | ARI | Silhouette Coefficient |
|---|---|---|---|---|
| Prong 1 | 99.80% | N/A | N/A | N/A |
| Prong 2 | 93.47% | 0.67 | 0.76 | 0.10 |
