# Supplementary figures and images for: Leveraging machine learning for taxonomic classification of emerging astroviruses
Source: Front Mol Biosci. 2024 Jan 11;10:1305506. doi: 10.3389/fmolb.2023.1305506 (PMC10808839; doi:10.3389/fmolb.2023.1305506)

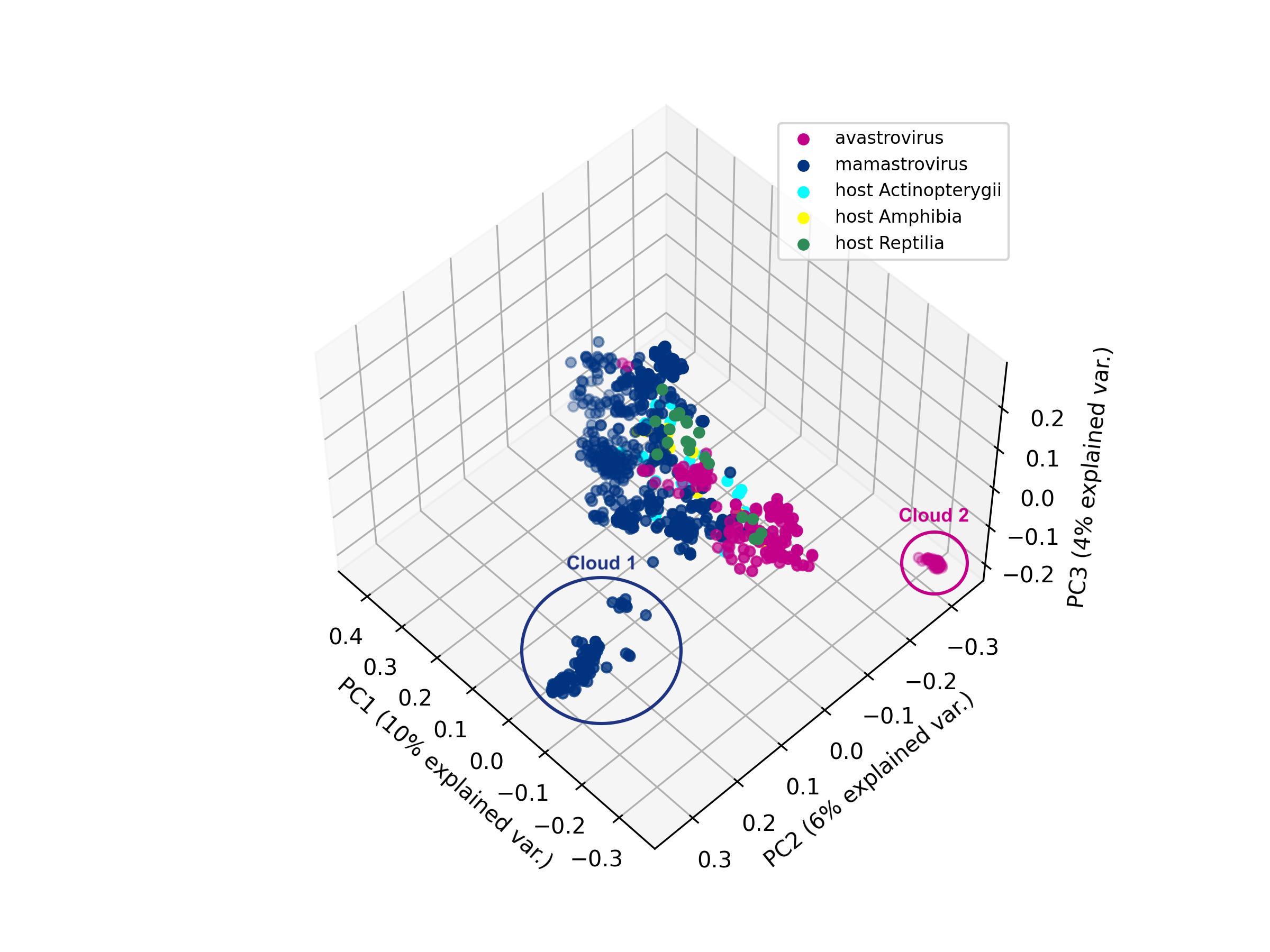

Supplement: Supplementary file 3 [file Image1.JPEG]

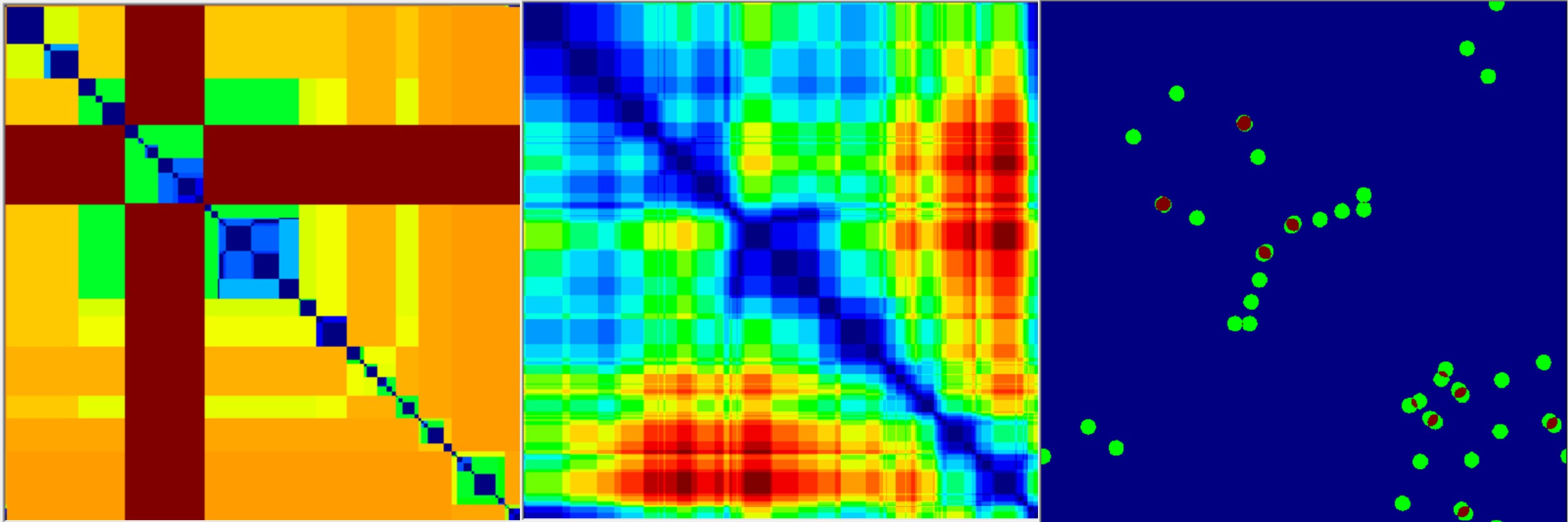

Supplement: Supplementary file 4 [file Image2.JPEG]
