# Supplementary material for: Leveraging machine learning for taxonomic classification of emerging astroviruses
Source: Front Mol Biosci. 2024 Jan 11;10:1305506. doi: 10.3389/fmolb.2023.1305506 (PMC10808839; doi:10.3389/fmolb.2023.1305506)
Supplement: Supplementary file 5 [file DataSheet3.pdf]

Supplementary Material 3: Performance Results of 3PCM Using Different Classification/Clustering Algorithms

1 PRONG 1 (SUPERVISED LEARNING)

Table S1 presents the performance results in terms of classification accuracy of fifteen algorithms as Prong 1 of 3PCM. In order to assess the accuracy of the classifiers, we used Stratified 10-Fold Cross-Validation. In Stratified K-Fold Cross-Validation, the data is divided so that each fold has approximately the same proportion of instances of each target class as the entire dataset. This is particularly important in the case of imbalanced datasets, where one class may have a much smaller representation than another (see Table 2 of the manuscript). We conducted 10 independent experiments for each classifier, considering one dataset partition as testing data and nine as training data. We then calculated the average of ten accuracies from each experiment and reported the results in Table S1.

Table S1. Classification accuracy of fifteen classifiers using a 10-fold cross-validation technique. The values in this table are averages for the use of 10 different validation datasets. As the results show, Quadratic SVM and Cubic SVM are the most accurate classification models for classifying astrovirus whole genomes among the candidates used.

Classifier	Classification Accuracy
10-Nearest Neighbours	99.12%
Nearest Centroid Mean	97.51%
Nearest Centroid Median	96.05%
Logistic Regression	98.54%
Linear SVM	98.97%
**Quadratic SVM**	**99.56%**
**Cubic SVM**	**99.56%**
SGD	99.41%
Decision Tree	97.80%
Random Forest	98.24%
AdaBoost	98.97%
Gaussian Naive Bayes	97.22%
LDA	90.23%
QDA	56.59%
Multilayer Perceptron	68.86%

2 PRONG 2 (UNSUPERVISED LEARNING)

The performance results of the clustering of the three clustering algorithm candidates measured in terms of the internal and external evaluation metrics are shown in Table S2.

Since K-means++ and GMM are non-deterministic algorithms and their outcomes can vary depending on the initialization parameters, we repeated both experiments twenty times with different initializations. The results shown in Table S2 represent the average of these twenty runs.

Table S2. Performance of Prong 2 for clustering DNA sequences of the *Astroviridae* family, with available taxonomic labels at the genera level, by utilizing three algorithms: K-Means++, GMM, and Hierarchical Clustering We employed classification accuracy, NMI [-1,1], ARI [-1,1], and silhouette coefficient [0,1] as evaluation metrics.

Clustering Algorithm	Classification Accuracy	NMI [-1,1]	ARI [-1,1]	Silhouette Coefficient [0,1]
**k-means++**	**88.16%**	**0.45**	**0.58**	**0.08**
GMM	70.47%	0.15	0.17	0.07
Hierarchical Clustering	78.51%	0.27	0.26	0.07

As part of our analysis of the dataset, we tested a number of linkage methods for Hierarchical Clustering. Ward's method provided the most coherent clustering and the highest silhouette coefficient score. Therefore, we have presented only the results of this linkage method in the table. We cut the Hierarchical Clustering tree at a suitable height in order to form exactly two clusters.

3 SELECTION OF k-MER SIZE

The performance of Prong 1 (quadratic SVM) and Prong 2 (K-means++) for different values of k for k-mer size in the range $[1, 9]$ in terms of classification accuracy and running time is shown in the Figure S1. As the figure shows, for $k = 6$ we reach the optimal performance in both prongs.

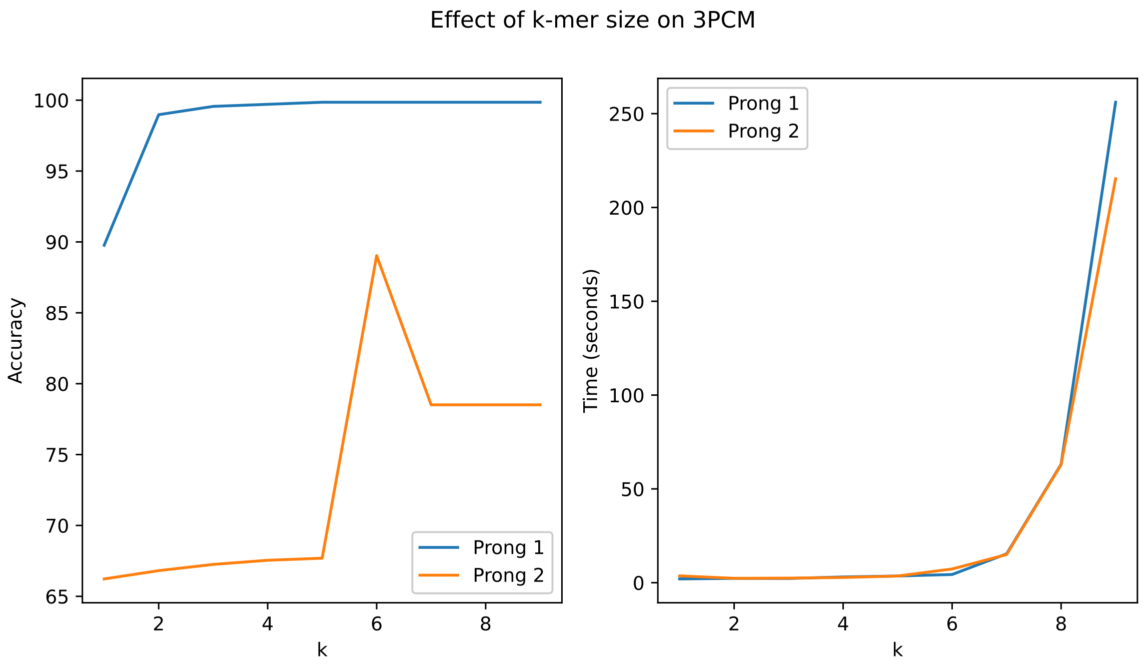

Figure S1. Effect of k-mer size in the performance of 3PCM's Prong 1 and Prong 2 in terms of classification accuracy (left panel) and running time (right panel).
