# Supplementary material for: Leveraging machine learning for taxonomic classification of emerging astroviruses
Source: Front Mol Biosci. 2024 Jan 11;10:1305506. doi: 10.3389/fmolb.2023.1305506 (PMC10808839; doi:10.3389/fmolb.2023.1305506)
Supplement: Supplementary file 6 [file DataSheet1.PDF]

Supplementary Material 1: Analysis of Astroviruses of Unknown Genus Label

A summary of the DNA sequence information for 308 as yet unclassified astrovirus genomes is shown in Table S1. NCBI categorizes these sequences as unknown at the genus level of their taxonomy classification.

Table S1. Distribution of the hosts of the 308 astrovirus genomes in Dataset 1 that are as yet unclassified at the genus level. Host labels are at the class level.

Host	No. of sequences	Min. seq. len.(bp)	Avg. seq. len.(bp)	Max. seq. len.(bp)
Amphibia	11	5,038	6,746	7,723
Arachnid	1	6,943	6,943	6,943
Aves	42	5,084	6,806	8,417
Actinopterygii	20	5,518	6,748	7,538
Chondrichthyes	4	6,450	6,951	7,168
Bivalvia	4	6,842	7,059	7,436
Insecta	4	5,243	5,476	5,832
Crustacea	5	5,030	5,834	6,984
Magnoliopsida	3	7,755	7,782	7,836
Sarcopterygii	2	6,865	6,927	6,989
Mammalia	187	5,209	6,348	7,426
Cephalaspidomorphi	4	6,555	7,148	7,642
Reptilia	21	5,062	7,188	8,840
All/Average	308	5,030	6,536	8,840

1 CLASSIFICATION OF AS YET UNCLASSIFIED ASTROVIRUSES WITH OTHER THAN MAMMALIAN AND AVIAN HOSTS

The genomes of as yet unclassified astroviruses with hosts other than Mammals and Avians were examined to determine whether they all belong to one of the two genera Mamastrovirus and Avastrovirus or if more than two genera may exist within this family of viruses. Accordingly, the hosts of 288 as-yet unclassified astroviruses presented in Table S1 were reviewed. There are fewer than six sequences available for each of the hosts Arachnida, Chondrichthyes, Bivalvia, Insecta, Crustacea, Magnoliopsida, Sarcopterygii, and Cephalaspidomorphi; these few sequences do not capture the variation of the Astrovirus genomes contributed by their hosts to the classification and clustering analysis. Although the minimum number of data points per cluster may differ depending on the complexity of the data and the clustering algorithm employed, a minimum of 10-20 data points per cluster is generally recommended to achieve reliable results in supervised and unsupervised machine learning Dalmaijer et al. (2022). Consequently, we decided to exclude the sequences belonging to these eight hosts and focus instead on the sequences belonging to the other five Animalia classes (Amphibia, Aves, Actinopterygii, Mammalia, and Reptilia).

In Section 3.2 of the manuscript, two host classes, Mammalia and Aves, were investigated and we succeeded to label most of these viruses as Mamastroviruses or Avastroviruses. For the purpose of investigating other as-yet unclassified Astroviruses, a new dataset was constructed consisting of 875

mammastrovirus and avastrovirus genomes labeled at the genus level (Dataset 2, comprising previously labeled mammastrovirus and avastrovirus genomes, as well as the 191 genomes with genus labels predicted by 3PCM), augmented with 11, 20, and 21 as yet unclassified astrovirus sequences with hosts belonging to the Amphibia, Actinopterygii, and Reptilia classes, respectively (see Table S2). This dataset was created to explore the separation of astroviruses with hosts other than Aves and Mammalia from each other and from mamastroviruses and avastroviruses. Prong 1 (supervised) was not applicable to this dataset, due to the absence of known astrovirus genomes with Amphibia, Actinopterygii, and Reptilia labels in the training set. The clustering results obtained by using Prong 2 (unsupervised) showed no clear separation between the as-yet unclassified astroviruses with hosts Amphibia, Actinopterygii, and Reptilia, nor was there any clear separation found between these genomes and avastroviruses and mamastroviruses.

In the absence of any conclusive clustering results, PCA was applied to this dataset, utilizing the first three principal components of the 6-mer counts for each genome, and preserving $\sim$20% of the explained variance. As seen in Figure S1, PCA also does not result in an obvious separation of the astrovirus genomes with hosts Amphibia, Actinopterygii, and Reptilia from each other, or from the Mamastrovirus and Avastrovirus sequence clusters. Additionally in this figure, Cloud 1 and Cloud 2 are shown representing a subset of mamastroviru and a subset of avastrovirus respectively. The accession IDs of the sequences in Clouds 1 and 2 are represented in Tables S3 and S4, respectively. In Section 3.3 of the main text, these two clouds of data points are explored.

Table S2. Description of the dataset including astrovirus genomes belonging to Avastrovirus and Mamastrovirus genera (previously taxonomically classified or the classification was proposed in Section 3.2 of the manuscript) augmented with astrovirus genomes of unknown genus and having one of the Reptilia, Actinopterygii, or Amphibia hosts.

Genus	Host	No. of sequences	Min. seq. len.(bp)	Avg. seq. len.(bp)	Max. seq. len.(bp)
Avastrovirus	-	239	5,130	7,113	8,417
Mamastrovirus	-	636	5,003	6,385	7,426
Unknown	Reptilia	21	5,062	7,188	8,840
Unknown	Actinopterygii	20	5,518	6,748	7,538
Unknown	Amphibia	11	5,038	6,746	7,723
All	-	927	5,003	6,599	8,840

REFERENCES

Dalmaijer, E. S., Nord, C. L., and Astle, D. E. (2022). Statistical power for cluster analysis. *BMC bioinformatics* 23, 1–28

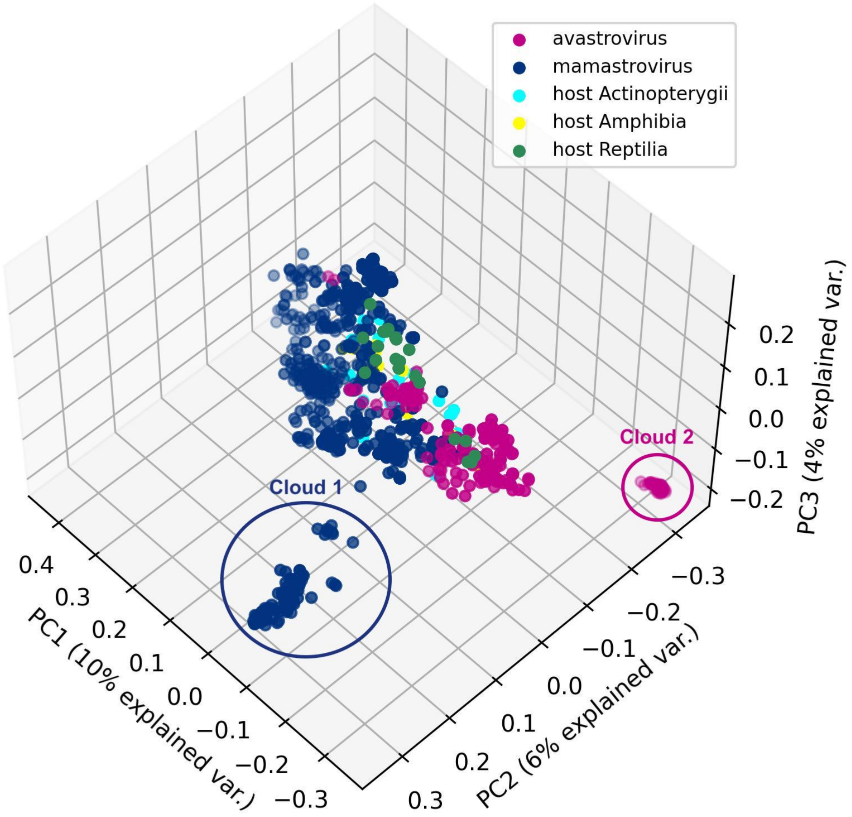

Figure S1. A comparison of as yet unclassified sequences obtained from hosts Amphibia, Actinopterygii, and Reptilia with 875 Astrovirus sequences belonging to Mamastrovirus or Avastrovirus. The visualization is based on the first three principal components of the 6-mer counts for the entire genome. In the figure, Cloud 1 represents a subset of mamastroviruses, while Cloud 2 represents a subset of avastroviruses.

Table S3. Accession IDs of the sequences in Cloud 1, representing a subset of mamastroviruses.

AY720892	GU732187	KF039912	MN433705	LC694991	MG571777	JN887820
MT906855	MN433703	MW485039	MZ603074	LC694985	GQ495608	MT906853
MZ603079	MW485038	DQ070852	KF039913	MT832893	MT832892	KC285113
MK059949	MW485041	MT832895	KC285152	FJ375759	DQ028633	JF491403
MK296753	LC694994	MN433704	LC694995	KY271945	MT267482	KY294673
LC694987	AB308374	MN444721	MF684776	MH446377	KF039910	LC694996
MN433706	MT267483	LC694988	MT906859	MW485040	MK059952	JF491430
AF260508	MH933759	MZ546174	Z25771	LC694990	MW485043	HQ398856
MT267476	MH933758	NC$_0$01943	MK059954	AY720891	MG921619	MH933752
MT832896	KC342249	MT906858	MG932587	KF039911	LC694989	MK059951
JF327666	HUANSSPS	MK618656	MW485042	MW485044	NC$_0$30922	MT832894
JQ403108	MT267480	MT906854	LC694992	MT832897	MH933756	KY271946
LC694993	FJ755404	MZ603072	MW485045	MH933757	GQ901902	ATVPOLY6A
AF141381	DQ344027	KF157967	MH332781	KP862744	MW863310	MT267478
MK059956	LC694986	MT906857	MT906856	LC694997		

Table S4. Accession IDs of the sequences in Cloud 2, representing a subset of avastroviruses.

KY807085	MF772821	MG934571	MH052598	MH807626	MK125058	MN068023
MN068024	MN103532	MN109954	MN109955	MN109956	MN109957	MN127951
MN127952	MN127953	MN127954	MN127955	MN127956	MN127957	MN127958
MN127959	MN175321	MN307114	MN307115	MN307116	MN307117	MN307118
MN307119	MN307120	MN337323	MN399857	MN428641	MN428642	MN428643
MN428644	MN428645	MN809622	MN894548	MT708902	MT934437	MT934438
MT934439	MW345727	MW413813	MW536497	MW592377	MW592378	MW592379
MZ367612	MZ540211	MZ576222	MZ819183	MZ819184	OK148600	OK571389
OK571390	OM273302	OM273303	OM273304	OM273305	OM273306	OM273307
OM273308	OM273309	OM273310				
