# Supplementary material for: Leveraging machine learning for taxonomic classification of emerging astroviruses
Source: Front Mol Biosci. 2024 Jan 11;10:1305506. doi: 10.3389/fmolb.2023.1305506 (PMC10808839; doi:10.3389/fmolb.2023.1305506)
Supplement: Supplementary file 7 [file DataSheet5.PDF]

Supplementary Material 5: Proposed Classification for as yet Unclassified Astroviruses

The following tables list the accession IDs of as-yet unclassified astroviruses for which a candidate genus assignment has been made by 3PCM. A classification label was suggested at the genus level for those sequences where all three prongs or only Prong 1 and Prong 2 are in agreement. The first section presents the results with the inclusion of candidate recombinant sequences in the dataset and the second section presents the results for the dataset with the exclusion of candidate recombinant sequences from the dataset. The teal color in the following tables represents accession IDs that are not in common between two sets of results derived from analyses that include and exclude recombinant sequences.

1 3PCM'S PROPOSED CLASSIFICATION WITH INCLUSION OF CANDIDATE RECOMBINANTS

Table S1. Accession IDs of the proposed avastroviruses when all three prongs agreed on the classification.

MK096774	MN920664	MN920670	MT137997	MT138004	MT138016	MN920669
MK096775	MN920665	MT137991	MT137999	MT138008	MT138014	MN920663
MK096776	MN920666	MT137992	MT138000	MT138009	MT138002	MT137994
MN920667	MT138013	MT138001	MT137993	MK450332		

Table S2. The Accession IDs of the proposed avastroviruses when there was agreement between Prong 1 and Prong 2 on the classification, but they disagreed with Prong 3.

JN420353	MH933754	NC_035758

Table S3. Accession IDs of the proposed mamastroviruses when all three prongs agreed on the classification.

AF141381	KU318319	LC549662	MN503236	MZ546174	OM140654	MN503235
FJ375759	KU318320	LC577870	MN503237	MZ603072	OM451104	MZ357117
L13745	KU318321	LC577871	MN503238	MZ603074	OM451105	NC_035471
JA816485	KX266901	LC577872	MN780842	MZ682112	OM451106	LC201619
JF327666	KX266902	MF175073	MN832787	MZ779126	OM451107	KU318317
JF713711	KX266903	MF175075	MN837531	MZ819166	OM451109	MZ218054
JN420351	KX266904	MG660832	MN837532	MZ819167	OM451110	LC201618
JN420352	KX266905	MG693175	MN837533	MZ819168	OM451111	KU318316
JN420353	KX266906	OM480543	MT152345	MZ819169	OM451112	MZ218053
JN420354	KX266907	MG921619	MT267478	MZ819170	OM451113	LC201617
JN420355	KX266908	MH933752	MT470220	MZ819171	OM451114	KU318315
JN420356	KX907127	MW863310	MT499771	MZ819172	OM451115	OK107512
JN420357	KX907128	MH933756	MT499772	NC_001943	OM451116	KY271945
JN420358	KX907129	MH933757	MT549856	NC_018702	OM451117	KM035759
JN420359	KX907130	MH933758	MT549857	NC_019027	OM451148	NC_037655
JX544743	KX907131	MH933759	MT642595	NC_023636	OM451210	MK404648
JX544744	KX907132	MK387176	MT734809	NC_024498	OM451211	KJ571486
JX556693	KX907133	MK395165	MT766313	NC_032423	OM480521	NC_035758
JX857869	KX907134	MK395166	MW082586	NC_032426	OM480533	KC692365
OM104033	LC201620	OK107515	MN087316	KU318318	OK107514	MK521913
ON571622	OK107513	MK521912	ON482290	KY271946	MZ005893	LC201616
OM890931	MW897147	LC201615	OM480545	MW897146	KF233994	OM480534
KC609001	KX907135	MK404645	MW249010	NC_032484	MZ357116	MH933753
MK404647	MK404649	MK404646	MG693176			

Table S4. The Accession IDs of the proposed mamastroviruses when there was agreement between Prong 1 and Prong 2 on the classification, but they disagreed with Prong 3.

KP663426	MT138010	NC_027426	ON304005	MK096773

2 3PCM'S PROPOSED CLASSIFICATION WITH EXCLUSION OF CANDIDATE RECOMBINANTS

Table S5. Accession IDs of the proposed avastroviruses when all three prongs agreed on the classification.

MK096774	MN920664	MN920670	MT137997	MT138004	MT138016	MN920669
MK096775	MN920665	MT137991	MT137999	MT138008	MT138014	MN920663
MK096776	MN920666	MT137992	MT138000	MT138009	MT138002	MT137994
MN920667	MT138013	MT138001	MT137993	MT138006		

Table S6. The Accession IDs of the proposed avastroviruses when there was agreement between Prong 1 and Prong 2 on the classification, but they disagreed with Prong 3.

JN420353	MH933754	NC_035758

Table S7. Accession IDs of the proposed mamastroviruses when all three prongs agreed on the classification.

AF141381	KU318319	LC549662	MN503236	MZ546174	OM140654	MN503235
FJ375759	KU318320	LC577870	MN503237	MZ603072	OM451104	MZ357117
HUANSSPS	KU318321	LC577871	MN503238	MZ603074	OM451105	NC_035471
JA816485	KX266901	LC577872	MN780842	MZ682112	OM451106	LC201619
JF327666	KX266902	MF175073	MN832787	MZ779126	OM451107	KU318317
JF713711	KX266903	MF175075	MN837531	MZ819166	OM451109	MZ218054
JN420351	KX266904	MG660832	MN837532	MZ819167	OM451110	LC201618
JN420352	KX266905	MG693175	MN837533	MZ819168	OM451111	KU318316
JN420353	KX266906	OM480543	MT152345	MZ819169	OM451112	MZ218053
JN420354	KX266907	MG921619	MT267478	MZ819170	OM451113	LC201617
JN420355	KX266908	MH933752	MT470220	MZ819171	OM451114	KU318315
JN420356	KX907127	MW863310	MT499771	MZ819172	OM451115	OK107512
JN420357	KX907128	MH933756	MT499772	NC_001943	OM451116	KY271945
JN420358	KX907129	MH933757	MT549856	NC_018702	OM451117	KM035759
JN420359	KX907130	MH933758	MT549857	NC_019027	OM451148	NC_037655
JX544743	KX907131	MH933759	MT642595	NC_023636	OM451210	MK404648
JX544744	KX907132	MK387176	MT734809	NC_024498	OM451211	KJ571486
JX556693	KX907133	MK395165	MT766313	NC_032423	OM480521	NC_035758
JX857869	KX907134	MK395166	MW082586	NC_032426	OM480533	KC692365
OM104033	LC201620	OK107515	MN087316	KU318318	OK107514	MK521913
ON571622	OK107513	MK521912	ON482290	KY271946	MZ005893	LC201616
OM890931	MW897147	LC201615	OM480545	MW897146	KF233994	OM480534
KC609001	KX907135	MK404645	MW249010	NC_032484		

Table S8. The Accession IDs of the proposed mamastroviruses when there was agreement between Prong 1 and Prong 2 on the classification, but they disagreed with Prong 3.

KP663426	MT138010	NC_027426
